# Recent innovations in fertilization with treated digestate from food waste to recover nutrients for arid agricultural fields

**DOI:** 10.1007/s11356-023-31211-2

**Published:** 2023-12-05

**Authors:** Dawid Skrzypczak, Krzysztof Trzaska, Małgorzata Mironiuk, Katarzyna Mikula, Grzegorz Izydorczyk, Xymena Polomska, Jerzy Wiśniewski, Karolina Mielko, Konstantinos Moustakas, Katarzyna Chojnacka

**Affiliations:** 1 Department of Advanced Material Technologies, Wroclaw University of Science and Technology, Lower Silesia, 50-370 Wroclaw, Poland; 2 Department of Biotechnology and Food Microbiology, Wroclaw University of Environmental and Life Sciences, Lower Silesia, 51-630 Wroclaw, Poland; 3 Department of Biochemistry, Molecular Biology and Biotechnology, Faculty of Chemistry, Wrocław University of Science and Technology, Łukasiewicza 2, 50-371 Wrocław, Poland; 4 School of Chemical Engineering, National Technical University of Athens, 9 Iroon Polytechniou Str., Zographou Campus, GR-15780 Athens, Greece

**Keywords:** Sustainable agriculture, Nutrient recovery, Micronutrients, Free amino acids, Waste valorization, Biogas digestate, Yeast waste

## Abstract

This study aims to explore the development of sustainable fertilizers from waste materials of a biogas plant and a brewery. These wastes, rich in organic carbon and nitrogen, were processed with sulfuric(VI) and phosphoric(V) acid mixture, facilitating the production of free amino acids and achieving waste sanitization. This treatment produced by-products, which extended the range of possible applications. The highest concentration of free amino acids (360 mg/l) was achieved through hydrolyzing with a 40% concentration medium over 24 h. In this case, the maximum levels were recorded for beta-alanine (69.3 mg/l), glycine (46.8 mg/l), isoleucine (43.5 mg/l), proline (36.2 mg/l), and valine (31.5 mg/l). The study presents two fertilizer technologies, with and without micronutrients, that satisfy European Parliament Regulation 2019/1009 (N_tot_ > 2%, N_org_ > 0.5%, C_org_ > 3%). Bioavailability of nutrients in the formulations ranged from 60 to 100%. The efficacies of these fertilizers were evaluated in 30-day pot trials with various plant species, with both single application and fertigation tested. Multielement analysis confirmed high nutrient transfer in the soil-plant system, and the inclusion of micronutrients led to biofortification of plant biomass in Cu (48.3 ± 7.2 mg/kg), Mn (249 ± 37 mg/kg), Zn (164 ± 25 mg/kg), and Fe (211 ± 32 mg/kg). These sustainable fertilizers present an alternative to traditional, non-renewable fertilizers and offer promising solutions for precision agriculture and environmentally conscious production.

**Graphical abstract:**

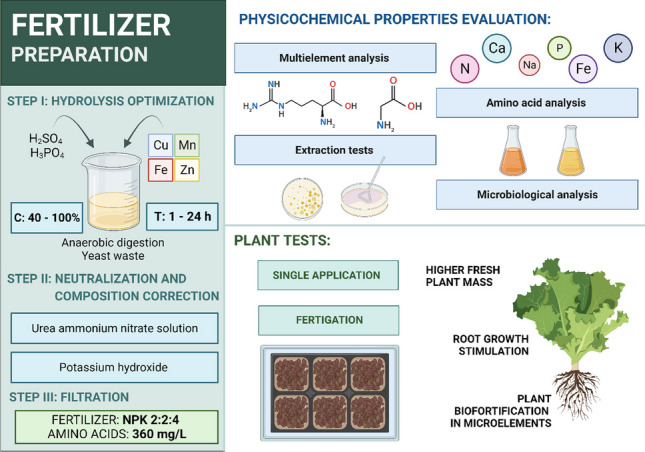

**Supplementary Information:**

The online version contains supplementary material available at 10.1007/s11356-023-31211-2.

## Introduction

The valorization of waste for fertilizer purposes is becoming the subject of extensive research by scientists around the world. The concept of this activity is to have the possibility to use the raw material potential of waste and through material recycling turn it around to the process loop (Bouhia et al. 2022). Biowaste, characterized by high organic matter content and low levels of contaminants, is particularly suitable for this goal (Fritsch et al. 2017; Mirabella et al. 2014). With proper processing of these wastes, the global volume is reduced, and valuable biogenic elements such as nitrogen, phosphorus, organic carbon, and potassium are recovered and reintroduced into the biocycle. Furthermore, through such actions, agriculture begins to be less intensive and thus more environmentally friendly (Megevand et al. 2022). This method presents an integrated and sustainable approach that aligns with the principles of circular economy, maximizing the value and utility of resources by their continual reuse and recycling.

An interesting group of wastes with high fertilizer potential are residues from the anaerobic digestion process (Barampouti et al. 2020). This technique aims to convert biomass, such as crop residues (Bedoić et al. 2019), animal manure (Holm-Nielsen et al. 2009), expired food (Pietrzyk and Klepacz-Smółka 2018), residues from agro-food processing (Mirabella et al. 2014), or sewage sludge (Xie et al. 2021), with the participation of microorganisms. AD results in the production of biogas, which is a mixture of methane and carbon dioxide with small additions of hydrogen, carbon monoxide, or water steam (Meegoda et al. 2018). Since methane, hydrogen, and carbon monoxide can be burned or oxidized, biogas can be used as fuel. In addition, anaerobic digestion is considered as energy recycling. However, it should be noted that the AD process is not waste-free. The resulting residues are still a burden on the environment and should be managed according to the idea of sustainability (Lamolinara et al. 2022).

The residues produced in the anaerobic digestion process are referred to as digestate, and their form and composition depend on the input material. Regardless of whether the digestate is liquid or solid form, it cannot be applied directly in agriculture. A high content of microorganisms is observed in the digestate mass, which would risk infecting plant crops, which are often consumed without any processing (Baştabak and Koçar 2020). However, digestate is a carrier of valuable elements, especially nitrogen and organic carbon, which are essential at each stage of plant growth (Risberg et al. 2017). As a valuable material, the digestate can be successfully processed and valorized into an agriculturally useful form, including fertilizers and soil improvers. These recycled products can play a vital role in ensuring nutrient availability, soil fertility, and overall sustainability in the agricultural sector. This will close the process loop, and the costly element in agriculture, nitrogen, will be safely utilized in farming (Monfet et al. 2017).

The composition of the digestate depends on the input material for the anaerobic digestion process (Czekała et al. 2022). Several digestates have been characterized in the literature, both in terms of macronutrient and micronutrient content. The nitrogen content varies between 0.06 and 1.24% d.m. if formed from food waste, 0.21–7.8% d.m. from the organic fraction of municipal waste, 0.14–2.1% d.m. from agricultural waste, or 0.05–0.62% d.m. from manure. The content of organic matter can reach up to 75% d.m. The digestates are also carriers of insignificant amounts of phosphorus up to 2.4% d.m., potassium up to 4.0% d.m., and other macronutrients like sulfur (up to 0.41% d.m.), magnesium (up to 0.51% d.m.), or calcium (up to 2.5% d.m.) (Risberg et al. 2017). Therefore, it can be concluded that the digestate contains all the macronutrients necessary for plants. Furthermore, it can also be a source of valuable micronutrients, especially iron, if anaerobic digestion of municipal wastewater sewage sludge has been carried out (Wang et al. 2020a). Additionally, the digestate can contain trace amounts of heavy metals, depending on the input material. Therefore, it is important to assess the potential heavy metal content to ensure the safety and sustainability of the recycled products.

Similarly to composition, digestate management methods depend on the input material and its form (Czekała et al. 2022). If the digestate occurs in solid form, it is most often processed through open or closed storage (Li et al. 2018) or composting (Manu et al. 2021). However, this involves large losses of nitrogen, environmental pollution through greenhouse gas emissions and leachate, and the still unresolved problem of residual waste. Rarely, solid digestate is used, such as sewage sludge, for the production of concrete elements, bricks, or road aggregate, although this reduces the strength parameters of the products (Hurst et al. 2023). Solid digestate is also considered an attractive soil-improving additive, but due to its high microbial content, it must undergo prior sterilization or hygienization (Fernández-Bayo et al. 2017). Liquid digestate is most often used agriculturally, although due to the presence of microorganisms and the low content of plant nutrients, it must be valorized (Vázquez-Rowe et al. 2015). Among the most common methods are acid or alkaline hydrolysis, used as a liquid additive in the production of solid fertilizers, concentration by evaporation, or recovery of valuable components using membrane technologies (Monlau et al. 2015). By using the above processes, liquid or suspension fertilizers that meet all the quality requirements for fertilizers under international laws and directives can be effectively obtained. An important added value is that liquid fertilizers can be successfully applied foliarly or in the soil through fertigation (Baştabak and Koçar 2020). While these management methods have their benefits, they also come with certain challenges. These include high energy consumption, greenhouse gas emissions, and the need for handling residual waste, which must be addressed to ensure the overall sustainability of the process.

**Fig. 1:**
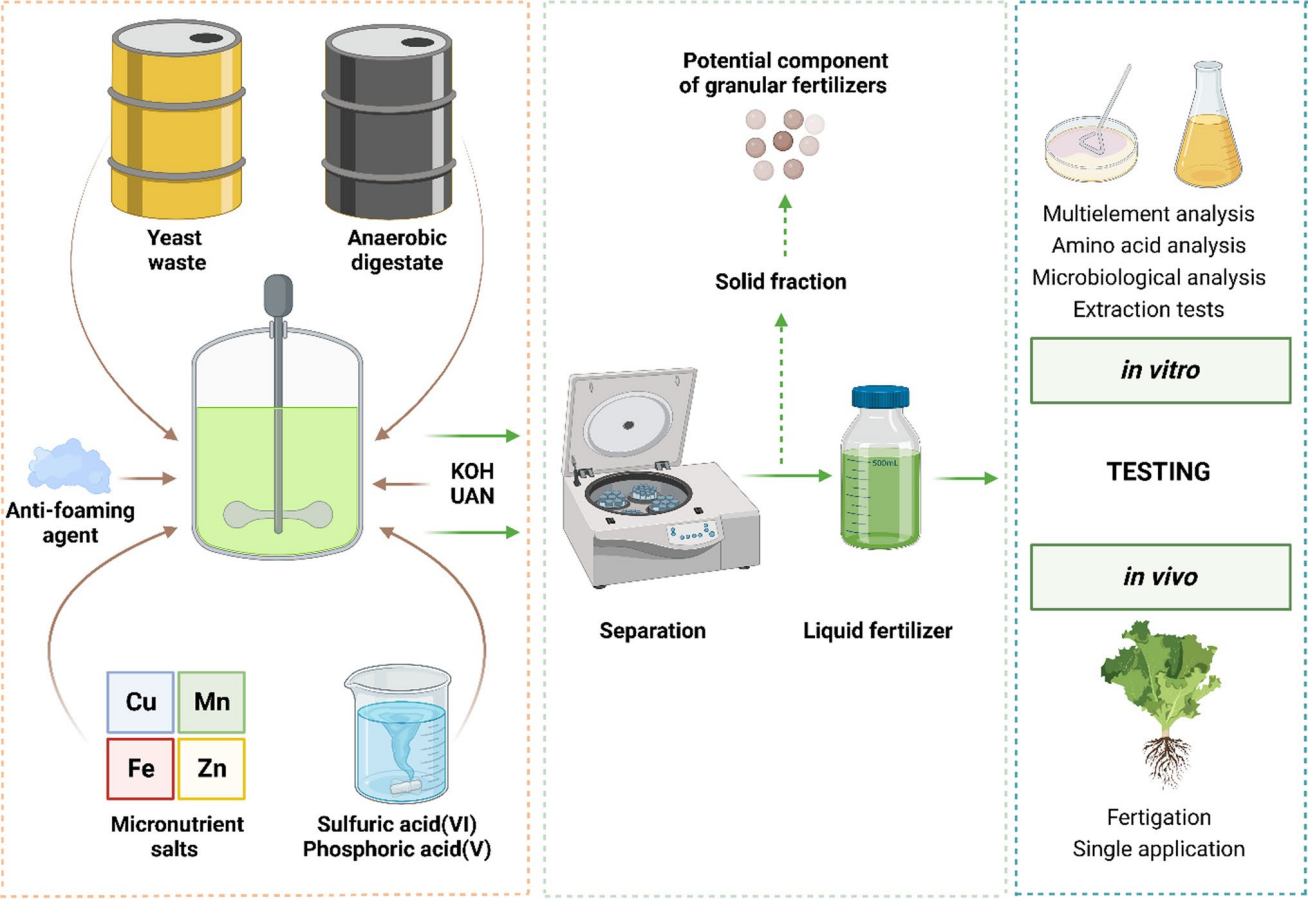
Schematic diagram of the production of liquid fertilizers based on anaerobic digestate. Created with BioRender.com

The following research presents the results of a study on the valorization of liquid digestate from food waste for fertilizer purposes. Through acid hydrolysis and compositional adjustment, two stable fertilizer formulations were obtained for use in fertilization. In the study, a full analysis of raw materials, intermediates, and fertilizers was carried out in terms of nutrient content and impurities, as well as microbiological analysis. Optimization of the hydrolysis process was performed on total amino acid content as a function of response. The resulting fertilizers were subjected to a biological efficiency evaluation in tests on a model plant — lettuce (*Lactuca sativa*) (Fig. 1). In this study, we used advanced analytical techniques, including inductively coupled plasma-optical emission spectrometry (ICP-OES), electrospray ionization mass spectrometry (ESI), atomic absorption spectroscopy (AAS), and thermoconductometric CN analysis to assess the nutrient content and impurities. Our findings offer new insights into the valorization of liquid digestate from food waste for fertilizer purposes, contributing to the body of knowledge in the field of sustainable agriculture and waste management. The study also compared the performance of different types of fertilization: single application and fertigation. The results were subjected to statistical analysis. The research conducted aims to increase the sustainability of agriculture and the waste treatment sector. It is in line with the line of countries in the action of European Union and other highly developed countries. The outcomes of this research are expected to provide practical insights for the development of efficient and sustainable strategies for the valorization of food waste digestate, thereby contributing to the advancement of sustainable agriculture and circular economy.

## Materials and methods

### Materials

All chemicals and reagents used were of analytical grade. The yeast waste (YW) used was from a local brewery. Anaerobic digestate (DS) from food waste was obtained from Bio-Wat, Świdnica (Poland). Yeast waste (YW) from a local brewery in Wroclaw (Poland) was used as an additional source of nitrogen. Hydrolysis was carried out using sulfuric acid(VI) (H_2_SO_4_) and phosphoric acid(V) (H_3_PO_4_) from STANLAB (Poland). Potassium hydroxide (KOH) was purchased from EUROCHEM BGD (Poland). Sulfate salts from Sigma-Aldrich (Poland) were used as sources of the trace elements: copper, iron, manganese, and zinc. Urea ammonium nitrate solution (UAN) from Grupa Azoty S.A. was used as a mineral nitrogen source.

### Process parameter selection

In nine beakers, 100 g each of DS and 130 g of YW were weighed as additional renewable nitrogen sources. To the prepared mixtures, 1 g of antifoaming agent (AA) and 60 ml of a mixture of sulfuric acid(VI) and phosphoric acid(V) (ratio 1.5:1) with a concentration ranging from 40 to 100% were added. The commercial product XIAMETERTM AFE-0310 was used as an antifoaming agent. It was selected because of its effectiveness at low concentrations (<1%) and ability to be used at pH below 3. The prepared samples were mechanically mixed over a time range of 1 to 24 h, at ambient temperature (Table 1). After the assumed time, 13 ml of UAN was added to the mixtures and neutralized with solid potassium hydroxide at pH ~ 5.0. The prepared samples were filtered and analyzed for free amino acids and multicomponent analysis. The selection of hydrolysis parameters was made on the basis of the highest concentration of free amino acids in the post-process solution.

**Table 1:** Variation of hydrolysis guidance parameters: optimization

Sample	DS	YW	AA	Acids *40% m/m*	Acids *60% m/m*	Acids *100% m/m*	UAN	KOH	Time
	*g*								*h*
1H40	100	130	1.00	60.0	-	-	13.0	19.7	1
1H60	100	130	1.00	-	60.0		13.0	32.9	1
1H100	100	130	1.00	-	-	60.0	13.0	57.7	1
6H40	100	130	1.00	60.0	-	-	13.0	20.3	6
6H60	100	130	1.00		60.0		13.0	32.1	6
6H100	100	130	1.00	-	-	60.0	13.0	57.9	6
24H40	100	130	1.00	60.0	-	-	13.0	20.4	24
24H60	100	130	1.00		60.0		13.0	31.2	24
24H100	100	130	1.00	-	-	60.0	13.0	56.1	24

### Application preparations

#### Fertilizer preparation

##### Formulation without micronutrients

The production process of the batch of liquid NPK fertilizer was carried out in an IKA LR1000 reactor with a mechanical turbine mixer. DS (300 g), YW (390 g) and AA (3 g) were placed in the reactor and then a mixture of sulfuric(VI) and phosphoric(V) acids (ratio 1.5:1) with a concentration of 40% was added. The hydrolysis process was carried out for 24 hours with constant stirring (75 Hz). After this time, 39 g of UAN was transferred to the hydrolysate, and then the mixture was neutralized to pH~5 with potassium hydroxide. The filtered solution was taken for further study (LF).

##### Formulation with micronutrients

The production process of the batch of application of liquid NPK fertilizer with micronutrients was carried out in an IKA LR1000 reactor with a mechanical turbine mixer. DS (300 g), YW (390 g), and AA (3 g) were placed in the reactor and then a mixture of sulfuric(VI) and phosphoric(V) acids (ratio 1.5:1) with a concentration of 40% was added. Next, the sulfate salts of copper (8.49 g), manganese (6.65 g), zinc (9.50 g), and iron (10.76 g) were transferred to the rector. The hydrolysis process in the presence of micronutrients was carried out for 24 h with constant stirring (75 Hz). After this time, 39 g of UAN was added to the hydrolysate, and then, the mixture was neutralized to pH~5 with potassium hydroxide. The filtered mixture was taken for further research (LFM).

#### Microbial analysis

Microbiological purity of raw materials (DS, YW) and liquid fertilizers (LF and LFM) was tested. Larger particles from DS and YW samples were removed by filtration through aseptic filtration assembly (Millipore) with a glass filter (Glass Fiber Filter Grade GA, pore size 1.6 μm, Frisenette). Permeate was used for further microbial analyses. In all samples, the total number of microorganisms (TNM), the total number of fungi including yeast and molds (TNF), as well as coliforms and *Escherichia coli* counts were determined. TNM and TNF were analyzed by pour plate methods on plate count agar (Merck, Millipore) and YGC agar (Millipore), respectively. Cultures were incubated at 28 °C for 72 h. Coliforms and *Escherichia coli* were counted on media pads *E. coli* and coliform (Millipore) after incubation at 37 °C for 24 h. Red-purple/navy colonies were identified as *E. coli*, while both red-purple/navy and green-blue/green colonies were counted as coliforms. The results from colony counts were given as colony-forming units per ml (CFU/ml). GLISA Singlepath Salmonella test (Millipore) was used for the analysis of the presence of *Salmonella* sp. Test was performed in accordance with the manufacturer’s instructions, and results were presented as the presence or absence of bacteria in 25 g of sample. All results were calculated from the analysis of five samples and represent arithmetic mean ± standard deviation.

#### Amino acid content

The samples were stored in 4 °C. Of each sample, 400 μl was centrifugated (14,000 rpm, 5 min, 4 °C). Next, 100 μl of supernatant was transferred to the new Eppendorf tube and extracted according to the MetAmino® Kit Manual procedure. In the first step, the microspin filter was activated: 200 μl microfilter solid-phase extraction (MSPE) sorbent activation medium (WES) was added and centrifugated for 30 s at 1500 ×g. After that, 200 μl of MSPE sorbent equilibration medium (EQS) was added and centrifugated under the same conditions. The solution which passed through was discarded.

##### Sample preparation

One hundred microliters of each sample and 100 μl of precipitation medium (PM) were added and centrifugated (1500 × g, 30 s). Twenty-five microliters was transferred to the new Eppendorf tubes, and internal standard (IS), a catalytic solution (CTS), and a reagent solution (RDS) were added. Between each reagent, the samples were mixed for 10 s. After that, the derivatization reaction was carried out for 2 min. In the next step, 400 μl of diluting and washing medium (DWM) was added, and the mixture was vortexed for 10 s. Of diluted reaction mixture, 450 μl was transferred to the wetted microspin filter sorbent. After 2 min, the samples were centrifugated in 1500 ×g for 30 s. Next, 200 μl of DWM was added and centrifugated under the same conditions. As the final step, the filters were transferred to the new Eppendorf tubes, and 200 μl of eluting medium (ELM) was added and centrifuged, as described previously. Of each sample, 150 μl was transferred into inserts.

For the measurements, the calibration procedure was also prepared. For the first calibration curve point, 20 μl of amino acid standard kit solution SD1 was mixed with 20 μl of amino acid standard kit solution SD2 and 10 μl of solution with internal standards (IS). The second point consisted of 10 μl of SD1, 10 μl of SD2, and 10 μl of IS. The third point included 10 μl of SD1, 10 μl of ten times diluted SD2, and 10 μl of IS. The fourth point involved 10 μl of 100 times diluted SD1, 10 μl of hundred times diluted SD2, and 10 μl of IS.

The prepared aliquots of the standard mixtures were processed according to the MetAmino® kit, as described above. The Synapt G2 Si Q-TOF mass spectrometer with an electrospray ion source (ESI) was combined with the Acquity UPLC I class chromatographic system (Waters, USA). The MassLynx software with the QuanLynx application (Waters, USA) was used for data processing and quantitative analysis. The samples were analyzed in positive polarity (ES+). The MS ion source parameters were set as follows: capillary voltage, 3.00 kV; sampling cone, 40 V; source temperature, 125 °C; desolvation temperature, 450 °C; nebulizer gas pressure, 5 bar; desolvation gas flow, 900 l/h; and cone gas flow, 50 l/h.

Chromatographic separation was performed on the analytical column (C18, 2.1 × 100 mm) included in the MetAmino® kit. The mobile phase consisted of 5 mM ammonium formate in water (A) and 5 mM ammonium formate in methanol (B). The flow rate was set at 0.300 ml/min and the total run time of the chromatographic method was 16 min. A gradient linear elution was performed according to the following steps: 0.50 min — 55% B; 10.00 min — 90% B; 10.50 min — 100% B; 12.00 min — 100% B; 12.01 min – 55% B. The autosampler and column temperature were set at 8 ° C and 35 °C, respectively. The sample volume injected into the column was 4 μl.

#### Chromium(VI) analysis

The chromium(VI) content of fertilizers was determined to assess compliance with the latest requirements of the European Union. A fertilizer sample, approximately 1g, was placed in a conical flask, followed by the addition of 100 ml of ultrapure water and shaking for 30 minutes. After the set time, the flask content was filtered. To the filtrate, 1 ml of concentrated zinc sulfate solution and a few drops of phenolphthalein were added, and then, the concentrated sodium hydroxide solution was gradually applied in portions until it became permanently pink. The obtained solution was filtered again, and the filtrate was subjected to ICP-OES analysis for chromium content.

#### Biuret analysis

The biuret content was determined by spectrophotometric method, according to the international standard PN-EN 15479. For this purpose, 10 g of fertilizer sample was dissolved in 250 ml of ultrapure water and filtered. In a 100-ml volumetric flask, 50 ml of the neutralized filtrate was placed, and 20 ml of alkaline potassium sodium tartrate solution and 20 ml of copper sulfate solution were added. The flask was refilled to 100 ml. If biuret is present in the sample, it forms a complex with copper ions, giving the solution a purple color. The intensity of the color is measured with a spectrophotometer at 546 nm. On the basis of the measured absorbance and the previously determined standard curve, the biuret content in the sample is calculated.

### Extraction test

#### Water-soluble micronutrients

The determination of water-soluble micronutrients was performed according to PN-EN 16962. LF (5 g) was transferred to a flask, and then, 250 ml of ultrapure water was added. The tightly closed vessel was shaken at 20 ± 2 °C for 60 min. After this time, the sample was filtered and subjected to multi-element analysis. The experiment was performed analogously for LFM. The concentration of water-soluble micronutrients was determined using Eq. 1:

1 $${C}_{F(i)}=\frac{C_{E(i)}\bullet {V}_E}{C_{m(i)}\bullet {m}_m}$$

where *C*_F(i)_ — form in fertilizer (%); *C*_E(i)_ — nutrient concentration in contact solution (mg/l); *C*_m(i)_ — nutrient concentration in extracted materials (mg/kg); *m*_m_ — mass of extracted materials (kg); *V*_E_ — extract volume (ml).

#### Water-soluble forms of macronutrients

The determination of the water-soluble calcium, sodium, magnesium and sulfur present in the form of sulfate content was performed according to PN-EN 15477. The LF sample (5 g) was transferred to a flask, and then, 400 ml of ultrapure water was added and boiled for 30 min. The sample was left at room temperature for 24 h. After this time, the sample was diluted to 500 ml, filtered, and subjected to multielement analysis. The experiment was performed analogously for LFM.

The extraction of water-soluble forms of phosphorus was performed according to EN 15958. The LF sample (5 g) was transferred to a flask, and 450 ml of ultrapure water was added. The solution was shaken for 30 min at 20 °C. After this time, the sample was diluted to 500 ml, filtered, and subjected to multielement analysis. The extraction was performed analogously for LFM. The concentration of water-soluble macronutrients was determined using Eq. 1.

#### Phosphorus forms soluble in neutral ammonium citrate

The extraction of soluble phosphorus forms in neutral ammonium citrate was carried out in accordance with PN-EN 15957. A sample of LF (1 g) was transferred to a flask containing 100 ml of neutral ammonium citrate previously heated to 65 °C. The sample was shaken at 65 °C for 1 h. After this time, the solution was cooled, filtered, and diluted to 500 ml. The prepared sample was subjected to multi-element analysis. The concentration of soluble phosphorus forms in neutral ammonium citrate was determined using Eq. 1.

### Plant tests

The conditions in the growth room were maintained at a temperature of 23 °C and a photoperiod of 16 h of light and 8 h of dark. Humidity control was not conducted. The fertilizers were applied manually with the pipette for each pot separately. Plant tests were carried out for a period of 4 weeks under artificial lighting conditions (2400 lux) in a dedicated growing room. Deacidified peat of pH 5.5–6 mixed with sand in a ratio of 8:2 v/v was used as the substrate. Multi-cells pots with the size of a single cell of 4.5 × 4.5 cm were filled with a mix of sand and peat. The lettuce seed (*Lactuca sativa*) was placed in each cell. The plants were watered with distilled water to maintain 70% soil moisture. Each cell with lettuce sprout was labelled and fertilized with the appropriate amount of RF, LF, and LFM fertilizers. Some of the plants were separated into a nonfertilized trial and watered only with water throughout the growing period. The tests were carried out at three doses for each fertilizer: 50%, 100%, and 150% of the optimal nitrogen dose per hectare. According to the publication (Sylvestre et al. 2019), the optimal dose of nitrogen that was selected for pot tests is 120 kg/ha. To achieve this goal, each plant was fertilized with 0.11 gN or 0.22 gN or 0.33 gN. After completion of the trials, the plants were manually removed from the soil. Roots were cleaned of soil residue and analyzed using WinRHIZO software (Regent Instruments Inc., Canada). Fresh weight was measured using a Sartorius-Quintix laboratory balance. Chlorophyll content was measured using a CCM300 instrument (Geomor-Technik, Poland) and expressed in milligrams per square meter.

#### Single application system

For a single fertilizer application, all scheduled RF, LF, and LFM fertilizers were applied on day 3 of the test. This avoided overfertilization of emerging seedlings. In the following days, the plants were watered only with deionized water.

#### Fertigation system

For fertilization in the form of fertigation, the appropriate dose of fertilizer corresponding to 60, 120, and 180 kgN/ha was diluted in 42 ml of deionized water. This solution was applied to the soil surface every 2nd day at a rate of 3 ml/plant. This amount of liquid was optimal to maintain proper soil moisture throughout the test period.

### Elemental composition analysis

The total content of nitrogen and carbon was determined using the thermoconductometric method TCD (CN Elementar Analyser, Vario MACRO Cube ELEMENTAR Analysensysteme, Germany). Samples of approximately 50 mg were placed in tin capsules and combusted at high temperature.

The mercury content was determined using AAS method with the amalgamation technique (AMA-254 mercury analyzer, ALTEC, Cech Republic). About 80–100 mg of the sample was weighed on nickel boats. The sample was dried and combusted in a stream of oxygen.

The content of the other macronutrients, micronutrients, and toxic elements was determined by the ICP-OES method (ICP-OES Varian Vista-MPX, Germany or iCAP 6500, Thermo Scientific, USA) after microwave-assisted digestion of the samples (START D Microwave Digestion System, Milestone, Italy). Samples of 0.5 g samples were mineralized in concentrated inorganic acids under appropriate time-temperature conditions. After mineralization, samples were diluted using ultrapure water to a mass of 50 g and subjected for multielement analysis after optimizing the spectrometer operating parameters. Calibration of the spectrometer was performed with compensation of matrix influences. This method is widely used in fertilizer analysis and is described as providing favorable results (Li et al. 2013).

The analysis of Cr(VI) content was performed according to the procedure described in the “Chromium(VI) analysis” section.

Each time, procedures were used to confirm the validity of the measurement results. They included the use of certified standards and matrix reference materials, the analysis of blank samples, and the testing of parallel samples. All measurements were performed in three replicates.

### Material balance

The material balance was based on direct measurements (mass of added materials). Elemental balance was done based on results from ICP-OES and thermoconductometric method TCD. Graphs were prepared in e!Sankey 5 software (IFU Hamburg GmbH, Germany). All results were converted per 1000 kg of final fertilizer (LF or LFM). Data are subject to 15% measurement uncertainty according to the ISO 17025 quality management system.

### Statistical analysis

Statistical analysis was performed using Statistica 13.3 software (TIBCO, USA). The first step of the analysis was to determine the normality of the distribution, which was done using the Shapiro-Wilk test (*p* > 0.05). Subsequently, in the case of a distribution non-normal, statistically significant differences were assessed using the Kruskal-Wallis test (*p* < 0.05). On the other hand, for a normal distribution of the results, the homogeneity of their variance was evaluated by the Brown-Forsythe test (*p* < 0.05). To assess statistically significant differences in results with homogeneous variances, Tukey’s RIR test was performed (*p* < 0.05), and in the case of heterogeneous variances, the Kruskal-Wallis test was used (*p* < 0.05).

## Results and discussion

### Raw material characterization

The raw materials used to produce the fertilizer were subjected to a chemical composition analysis to verify their suitability for fertilization. In the course of the study, the total content of macro and micro-nutrients, as well as toxic elements, was determined in the yeast waste and anaerobic digestate. The results of the analysis of the elemental composition are shown in Table 2. The physical properties of the raw materials are shown in Table S1.

**Table 2:** Multielemental analysis of raw materials

Samples	Macroelements							
	N	C	P_2_O_5_	K_2_O	CaO	MgO	SO_3_	Na_2_O
	*%*	*%*	*%*	*%*	*%*	*%*	*%*	*%*
DS	0.662 ± 0.067	1.51 ± 0.15	0.312 ± 0.047	0.198 ± 0.030	0.228 ± 0.034	0.0445 ± 0.0067	0.161±0.024	0.311±0.047
YW	2.200 ± 0.220	13.4 ± 1.3	0.584 ± 0.088	0.385 ± 0.058	0.207 ± 0.031	0.0603 ± 0.0090	0.270±0.041	<LOD
Samples	Microelements							
	Cu		Fe		Mn		Zn	
	*mg/kg*		*mg/kg*		*mg/kg*		*mg/kg*	
DS	8.01 ± 1.20		461 ± 69		5.21 ± 0.78		16.7 ± 2.51	
YW	14.4 ± 2.2		23.4 ± 3.5		5.31 ± 0.80		10.4 ± 1.56	
Samples	Toxic elements							
	As		Cd	Cr(VI)	Hg		Ni	Pb
	*mg/kg*		*mg/kg*	*mg/kg*	*mg/kg*		*mg/kg*	*mg/kg*
DS	0.306 ± 0.046		0.0913 ± 0.0137	<LOD	0.0250 ± 0.003		<LOD	<LOD
YW	0.471 ± 0.071		0.0287 ± 0.0043	<LOD	0.0062 ± 0.001		<LOD	<LOD

In the context of beer production, improper management of organic waste can lead to a variety of environmental challenges. In 2021 alone, EU countries are estimated to have produced a colossal 33.1 billion liters of beer, with Poland being the second-largest producer and contributing 11% of the total production (Eurostat 2022). This level of production inevitably leads to the generation of significant quantities of organic waste. The beer production process yields large amounts of spent grains, hops, and yeast, which, if not managed effectively, can have a substantial environmental impact. Incorrect disposal can lead to land and water pollution, while organic waste left to decompose may release potent greenhouse gases into the atmosphere. Improper waste handling may compromise operational efficiency in breweries and contribute to heightened production costs. If this waste stream is managed appropriately, it presents an opportunity for conversion into valuable by-products. Spent grains, for instance, can be repurposed into animal feed, compost, or bioenergy, among other applications. Therefore, the opportunity for more sustainable waste management practices in the beer production industry becomes evident. Their management in accordance with the principles of circular economy is a major challenge. Spent brewery yeast is one of the main by-products in the brewery industry (about 2.3 kg/m^3^ of beer) (Gokulakrishnan et al. 2023; Vieira et al. 2016). Currently, the main direction of their management is their use for feed purposes (Bonato et al. 2022). One of the alternatives is to use this waste as raw material for fertilizer production.

Analyses of the composition results of spent brewer yeast show that it contains valuable nutrients (Puligundla et al. 2020). The study confirmed that the waste yeast is a great source of N (2.2%) and C (13.4%), a good source of P (0.58% as P_2_O_5_), K (0.38% as K_2_O), which, on a dry weight basis, gives 10.18% N, 62.04% C, 2.68% P_2_O_5_, and 1.76% K_2_O, respectively. It also contains microelements — in milligrams per kilogram dry mass: Cu, 66.7; Fe, 2134; Mn, 24.6; and Zn, 48.1. In the literature, a similar composition of waste yeast is reported. Waste yeast (a by-product from beer production) predominantly consists of the yeast strain *Saccharomyces cerevisiae*, used in the brewing industry. The content values for elements such as nitrogen (N), carbon (C), phosphorus (P_2_O_5_), potassium (K_2_O), and trace elements, including copper (Cu), iron (Fe), manganese (Mn), and zinc (Zn), can be the source of fertilizer nutrients. The nutritional value of YW depends largely on the quality and raw material and the conditions of the process (Gokulakrishnan et al. 2023). The heavy metal content in the waste yeast was at very low levels. Furthermore, yeast shows high absorption properties to heavy metals (Bonato et al. 2022). Scientific papers describe their effectiveness in bioremediation processes (Daverey et al. n.d.). Due to its high content of protein, minerals, and vitamins, they can be a source of nutrients for microorganisms (Ferreira et al. 2010).

Studies show that currently only less than 6% of yeast is used for fertilizer purposes (Bonato et al. 2022). Given the current situation in the market for fertilizer raw materials and the composition of the renewable raw materials tested, the use of them for fertilizer production seems to be in the right direction. It is also beneficial to use biochar made from brewery yeast. It has a positive effect on phosphorus, potassium content in plants, and soil fertility by increasing carbon, nitrogen, and phosphorus contents (Manolikaki and Diamadopoulos 2020).

Due to its high nutrient content, the digestate is successfully used as an organic fertilizer in crop production, e.g., in the cultivation of solanaceous and leafy vegetables (Jin et al. 2022) and maize (Zilio et al. 2023), both in conventional and hydroponic cultivations (Södergren et al. 2022). These studies underscore the value of the digestate from waste yeast as a nutrient source, reinforcing its potential for sustainable agriculture.

The digestate used in our study contained essential nutrients for plants. In dry matter, the content of macronutrients was as follows: 1.79% N, 4.08% C, 0.84% P_2_O_5_, 0.54% K_2_O, 0.62% CaO, 0.12% MgO, and 0.44% SO_3_. Among micronutrients, Fe was the highest with a dry mass of 1246 mg/kg. The content of Cu, Mn, and Zn was comparable with that of waste yeast. The digestate contained a relatively high amount of Na, which may be due to the use of food waste as a raw material in the fermentation process. The specific values of Na content in the digestate used in the study are provided. While Na is not widely recognized as an essential element for plants, studies show that in certain situations where there is limited availability of K, Na can partially take over its functions (Santa-María et al. 2023). Although Na is not inherently harmful to plants, it can very easily reach toxic levels (Benito et al. 2014). Therefore, it is necessary to control the sodium content of the final product. Similarly, as with waste yeast, the heavy metal content was at a very low level.

A study on digestate use in the cultivation of eggplant and Shanghai cabbage showed that, when applied at the right dose, digestate has a positive effect on increasing yields and improving nitrogen use efficiency (Jin et al. 2022). Its effectiveness as a fertilizer in the production has also been confirmed (Hultberg et al. 2023). Additionally, due to the content of organic substances, it positively affects the physical and chemical parameters of the soil (Samoraj et al. 2022).

Elemental composition analyses have confirmed that both yeast and digestate can be a good source of fertilizer nutrients. By using digestate and yeast waste as fertilizer raw material, it is possible to recover and close the nutrient cycle.

### Process parameter selection

The hydrolysis process was determined by two variables: acid concentration (40% (m/m), 60% (m/m), 100% (m/m)) and the duration of the process (1 h, 6 h, and 24 h). The most favorable variant was considered the one in which the response function, i.e., the total amino acid content took the highest value. The decision to measure amino acids was primarily driven by their critical role in plant nutrition. Amino acids are the building blocks of proteins and are essential for plant growth and development. Certain amino acids can influence plant metabolic processes and stress responses. The amino acid content of the yeast waste has direct implications for its potential as an organic fertilizer. By assessing the total amino acid content in various conditions, we aimed to optimize the hydrolysis process for maximum nutrient release, aligning with the study objective of improving the value and efficacy of this brewing by-product. Twenty-one amino acids were analyzed. The results are summarized in Table 3. The contents of the components in the hydrolysate, solid, and liquid fractions after the process are presented in Table S2.

**Table 3:** Amino acid content of hydrolysates from parameter optimization

Compound	Concentration								
	*mg/l*								
	1H40	1H60	1H100	6H40	6H60	6H100	24H40	24H60	24H100
Aspartic acid	1.53 ± 0.05	1.62 ± 0.05	1.74 ± 0.05	2.98 ± 0.09	2.94 ± 0.09	2.31 ± 0.07	3.19 ± 0.10	3.48 ± 0.10	4.17 ± 0.13
Serine	5.74 ± 0.17	9.14 ± 0.27	4.30 ± 0.13	10.6 ± 0.3	7.01 ± 0.21	5.25 ± 0.16	17.3 ± 0.52	13.7 ± 0.4	6.59 ± 0.20
Histidine	0.212 ± 0.006	0.260 ± 0.008	0.140 ± 0.004	0.370 ± 0.011	0.220 ± 0.007	0.220 ± 0.007	0.570 ± 0.017	0.350 ± 0.011	0.230 ± 0.007
Arginine	2.81 ± 0.08	3.52 ± 0.11	2.54 ± 0.08	4.31 ± 0.13	3.43 ± 0.10	3.01 ± 0.09	5.77 ± 0.17	5.01 ± 0.15	3.54 ± 0.11
Threonine	5.85 ± 0.18	8.33 ± 0.25	4.87 ± 0.15	8.41 ± 0.25	5.89 ± 0.18	5.77 ± 0.17	14.5 ± 0.4	11.5 ± 0.3	6.13 ± 0.18
Beta-Alanine	43.6 ± 1.3	28.9 ± 0.9	39.0 ± 1.2	60.4 ± 1.81	45.2 ± 1.4	69.6 ± 2.1	69.3 ± 2.1	73.9 ± 2.2	71.6 ± 2.1
Proline	12.2 ± 0.4	16.7 ± 0.5	12.7 ± 0.4	19.1 ± 0.6	16.3 ± 0.5	15.7 ± 0.5	36.2 ± 1.1	31.7 ± 0.9	22.2 ± 0.7
Tyrosine	5.74 ± 0.17	5.85 ± 0.18	6.32 ± 0.19	8.63 ± 0.26	7.84 ± 0.24	7.95 ± 0.24	10.0 ± 0.3	9.97 ± 0.30	10.4 ± 0.3
Valine	13.3 ± 0.4	17.3 ± 0.5	14.6 ± 0.4	20.9 ± 0.6	19.0 ± 0.6	16.8 ± 0.5	31.5 ± 1.0	30.3 ± 0.9	24.3 ± 0.7
Methionine	6.00 ± 0.18	6.41 ± 0.19	6.90 ± 0.21	7.74 ± 0.23	7.04 ± 0.21	8.62 ± 0.26	11.4 ± 0.3	10.0 ± 0.3	9.04 ± 0.27
Isoleucine	18.6 ± 0.56	23.6 ± 0.7	22.7 ± 0.7	26.0 ± 0.8	24.0 ± 0.7	26.8 ± 0.8	43.5 ± 1.3	41.6 ± 1.2	34.7 ± 1.0
Leucine	12.8 ± 0.4	13.9 ± 0.4	14.3 ± 0.4	21.5 ± 0.6	21.0 ± 0.6	17.5 ± 0.5	23.1 ± 0.7	24.8 ± 0.7	28.4 ± 0.8
Phenylalanine	10.3 ± 0.3	9.56 ± 0.29	12.7 ± 0.4	15.2 ± 0.4	15.4 ± 0.5	15.4 ± 0.5	15.2 ± 0.7	16.5 ± 0.5	21.5 ± 0.6
Lysine	1.34 ± 0.04	2.56 ± 0.08	0.970 ± 0.029	3.12 ± 0.09	1.95 ± 0.06	1.30 ± 0.04	5.80 ± 0.17	4.51 ± 0.14	2.08 ± 0.06
Glutamine	0.173 ± 0.005	0.280 ± 0.010	0.100 ± 0.003	0.330 ± 0.010	0.190 ± 0.006	0.110 ± 0.003	0.360 ± 0.011	0.200 ± 0.006	0.0600 ± 0.0020
Tryptophan	6.67 ± 0.20	6.37 ± 0.19	8.67 ± 0.26	8.70 ± 0.26	8.68 ± 0.26	10.1 ± 0.3	9.32 ± 0.28	9.58 ± 0.29	11.3 ± 0.3
Asparagine	0.092 ± 0.003	0.160 ± 0.005	0.0800 ± 0.0024	0.220 ± 0.007	0.180 ± 0.005	0.100 ± 0.003	0.330 ± 0.010	0.250 ± 0.008	0.160 ± 0.005
Glycine	34.1 ± 1.0	22.0 ± 0.7	30.0 ± 0.9	42.3 ± 1.3	26.6 ± 0.8	54.3 ± 1.6	46.8 ± 1.4	38.1 ± 1.1	35.3 ± 1.1
Alanine	5.42 ± 0.16	7.02 ± 0.21	4.43 ± 0.13	9.34 ± 0.28	7.46 ± 0.24	5.73 ± 0.17	13.9 ± 0.4	11.1 ± 0.3	8.14 ± 0.24
Cystine	0.0460 ± 0.001	0.0600 ± 0.0018	0.0400 ± 0.0120	0.0800 ± 0.0024	0.0500 ± 0.0015	0.0500 ± 0.0015	0.150 ± 0.004	0.110 ± 0.003	0.0600 ± 0.0018
Beta-aminobutyric acid	0.262 ± 0.008	0.410 ± 0.012	0.290 ± 0.009	0.660 ± 0.020	0.540 ± 0.162	0.690 ± 0.021	1.22 ± 0.04	1.41 ± 0.04	2.29 ± 0.07
Total	187 ± 6	184 ± 5	187 ± 6	271 ± 8	221 ± 7	267 ± 8	360 ± 11	338 ± 10	302 ± 9

Based on the results, it was considered most favorable to run the hydrolysis for 24 h at 40% (m/m). The data in this study were analyzed using a methodical approach. Experiments were conducted at diversified process parameters: three levels of acid concentration (40%, 60%, 100%) and three different durations of hydrolysis (1 h, 6 h, 24 h). The total amino acid content was then measured in the resulting hydrolysate. The experiment yielding the highest concentration of total amino acids was deemed optimal, which in our case was the 24-h hydrolysis run at a 40% acid concentration, producing a total amino acid content of 360 mg/l.

After the hydrolysis process under the presented conditions, the parameters of liquid fertilizers change significantly. The viscosity of the raw digestate was 82.1 mPa*⋅*s (Table S1), and after the hydrolysis process, it was reduced to 39.7 for LFM and 61.8 for LF (Table S3). This change will reduce the workload of the pumps used to distribute it by also reducing energy use. The reduction in viscosity is related at the same time with a decrease in solid content (liquification in the hydrolysis process) which was enhanced by filtration. The resulting fertilizer was a cloudy liquid with no clearly visible solids. This form allows it to be distributed mechanically in the agricultural industry. Additional filtration of larger solids before application is not required, and clogging of sprayer nozzles is reduced.

To further our understanding, we observed how changing acid concentration and duration affected the hydrolysis process. An inverse relationship was observed between temperature and efficiency, with efficiency dropping as temperature rose. This trend is also documented in previously published work, confirming that amino acid degradation is indeed promoted by higher concentration of acids (Thakkar et al. 2022). Simultaneously, our findings showed that prolonging the duration yielded higher total amino acid content, indicating the advantages of a longer process time. It is crucial to note that both the characteristics of the material undergoing hydrolysis and the specific outcomes desired (the response function) play significant roles in determining the ideal conditions for hydrolysis. As pointed out in related research (Sajib et al. 2020), these factors can result in variations in the optimal hydrolysis conditions. The total amino acid content of this hydrolysate was 360 mg/l. On the analysis of the other samples, it can be concluded that increasing the temperature of hydrolysis decreases its efficiency; in turn, the running time should be as long as possible. Similar relationships have been reported in the literature, indicating that an increase in temperature affects the breakdown of amino acids (Chen et al. 2019). However, other studies have noted that the temperature of hydrolysis as well as its time should be adapted to the material and the expected response function (Sajib et al. 2020).

Of the amino acids analyzed, under the most optimal time-temperature regime, the highest content was recorded for beta-alanine (69.3 mg/l), glycine (46.8 mg/l), isoleucine (43.5 mg/l), proline (36.2 mg/l), and valine (31.5 mg/l). Prior to hydrolysis, the digestate exhibited a comparatively lower concentration of the key amino acids, with these increasing significantly post-process. This confirms the effectiveness of hydrolysis in releasing these vital amino acids from the complex organic matrix of the yeast waste. The rise in amino acids concentrations underscores the utility of hydrolysis in enhancing the bioavailability of these essential amino acids, thereby augmenting the biostimulant properties of the digestate for plant growth. Free amino acids play an important role in plant growth. They provide ready-made building blocks that can be rapidly incorporated into the metabolic pathways of plants. Thus, the plant does not need to synthesize them on its own. Therefore, amino acids are considered biostimulants for plant growth, improving plant vigor and yield. The aforementioned amino acids with the highest concentrations play a number of important roles. Beta-alanine is recognized as a compound that reduces stress associated with high temperature, drought, or heavy metal contamination of the soil (Parthasarathy et al. 2019). Glycine promotes the uptake and better utilization of fertilizer nutrients by plants, effectively reducing their dosage (Zargar Shooshtari et al. 2020). Isoleucine strengthens plant resistance against fungal diseases (Li et al. 2021). Proline reduces the content of reactive oxygen species, which delays aging and cell death (Ghosh et al. 2022). On the other hand, valine is responsible for strong rooting and growth, which increases the water-absorbing surface (Li et al. 2020).

### Application formulations

#### Nutrient balance

The final LF and LFM fertilizer formulation was based on digestion residues from an agricultural-food biogas plant and brewery waste yeast. These substrates were hydrolyzed using sulfuric(VI) and phosphoric(V) acids, which led to the inhibition of microbial activity and the fermentation process. This reduction of biological activity was crucial for limiting nitrogen emissions into the atmosphere and managing potential changes in the rhizosphere (Odlare et al. 2008).

Microorganisms play an essential role in the nitrogen cycle. Their activity, including nitrification and denitrification processes, was curtailed by acid hydrolysis. Consequently, this resulted in the immobilization of nitrogen within the substrate, preventing its escape as a gas. This controlled nitrogen volatility not only reduces environmental impact but also preserves nitrogen in the substrate, enhancing the fertilizer potential.

The use of strong inorganic phosphoric(V) and sulfuric(VI) acids shifted the substrate pH to acidic, enhancing the macronutrient content in the final product. The lower pH facilitated the solubilization of certain macronutrients present in the waste yeast, increasing their bioavailability. Furthermore, phosphoric and sulfuric acids contributed essential nutrients, phosphorus and sulfur, respectively, increasing the macronutrient content and suppressing microbial activity to prevent nitrogen volatilization.

The potassium content, expressed as P_2_O_5_, increased from 0.312% in the digestate to 2.53% and 2.16% in LF and LFM, respectively. Similarly, sulfur content, expressed as SO_3_, escalated from 0.161 to 2.81% in LF. Sulfur plays a critical role in plant growth, participating in the formation of proteins, amino acids, vitamins, and chlorophyll. The heightened sulfur content in our fertilizer enhances its efficacy. This sulfur content was further amplified to 3.18% in the LFM fertilizer with the addition of micronutrient additives in sulfate form.

Alongside with sanitization, our fertilizers also retained essential macronutrients, such as calcium (Ca), magnesium (Mg), and sodium (Na), in quantities akin to the raw digestate (Czekała et al. 2022). Both LF and LFM compositions met the European Parliament Regulation 2019/1009 for organic-mineral fertilizers, with nitrogen contents of 2.30% and 2.33% by weight, respectively (EUR-Lex 2023).

The new formulations also satisfied the requirements for multi-component organic-mineral fertilizers due to the content of P_2_O_5_ and K_2_O being >2% and their total quantity surpassing 6%. Both compositions fulfilled the regulation mandate for an organic carbon content of >3%. Toxic elements, such as As, Cd, Cr(VI), Hg, Ni, and Pb, did not exceed the permitted limits of 40, 3.0, 2.0, 1.0, 50, and 120 mg/kg d.w., respectively. Complying with these standards allows the LF and LFM fertilizers to be registered as fertilizer products in the EU and introduced to the market (Table 4). The physical properties of the raw materials are shown in Table S1.

**Table 4:** Multielemental analysis of application formulations

Sample	Macroelements							
	N	C	P_2_O_5_	K_2_O	CaO	MgO	SO_3_	Na_2_O
	*%*	*%*	*%*	*%*	*%*	*%*	*%*	*%*
LF	2.30 ± 0.23	5.56 ± 0.56	2.53 ± 0.38	5.94 ± 0.89	0.157 ± 0.024	0.0430 ± 0.0065	2.81 ± 0.422	0.140 ± 0,021
LFM	2.33 ± 0.23	4.84 ± 0.48	2.16 ± 0.32	5.02 ± 0.75	0.148 ± 0.022	0.0534 ± 0.0080	3.18 ± 0.477	0.134 ± 0,020
Sample	Microelements							
	Cu		Fe			Mn	Zn	
	*mg/kg*		*mg/kg*			*mg/kg*	*mg/kg*	
LF	6.12 ± 1.6		193 ± 28			8.87 ± 1.33	10.6 ± 1.59	
LFM	1751 ± 350		2056 ± 411			1741 ± 348	1806 ± 361	
Sample	Toxic elements							
	As		Cd	Cr(VI)	Hg	Ni	Pb	Biuret
	*mg/kg*		*mg/kg*	*mg/kg*	*mg/kg*	*mg/kg*	*mg/kg*	*g/kg*
LF	<LOD		0.272 ± 0.041	<LOD	0.0075 ± 0.001	<LOD	<LOD	0.012 ± 0.001
LFM	<LOD		2.31 ± 0.35	<LOD	0.0110 ± 0.001	<LOD	10.3 ± 1.5	0.014 ± 0.001
	Acceptable limits according to Regulation (EU) 2019/1009 of the European Parliament and of the Council for organic-mineral fertilizers							
	**≤40**		**≤3.0**	**≤2.0**	**≤1.0**	**≤50**	**≤120**	**≤12**

The multielemental analysis provided in Table 9 offers a comprehensive overview of macroelements, microelements, and toxic elements present in the LF and LFM application formulations. In terms of macroelements, both the LF and LFM samples contained nitrogen (N), carbon (C), phosphorus (P_2_O_5_), potassium (K_2_O), calcium oxide (CaO), magnesium oxide (MgO), sulfur trioxide (SO_3_), and sodium oxide (Na_2_O). Slight variations were observed in the concentrations of these elements between the LF and LFM samples. For instance, the LF sample showed slightly higher levels of carbon, phosphorus, and potassium, while the LFM sample demonstrated marginally increased concentrations of nitrogen, magnesium oxide, and sulfur trioxide. Considering microelements, a stark difference was observed between the LF and LFM samples. The LFM sample contained higher levels of Cu, Fe, Mn, and Zn when compared to the LF sample. Determination of toxic elements demonstrated a minimal presence of As, Cr(VI), Hg, Ni, and Pb in the LF and LFM samples. It was shown that cadmium (Cd) was low in both samples, but significantly higher in the LFM sample, compared to the LF sample. The analysis showed that the concentration of toxic elements in both the LF and LFM samples was within the acceptable limits according to Regulation (EU) 2019/1009 of the European Parliament and of the Council for organic-mineral fertilizers (EUR-Lex 2023). Cadmium in the LFM sample, though considerably higher than in the LF sample, still remained below the threshold value. The multielemental analysis indicates that both the LF and LFM formulations are rich in essential macro- and microelements while maintaining the presence of toxic elements well within the established safety limits. This suggests their potential effectiveness and safety as fertilizers in agricultural applications. Nonetheless, the significant variation in elemental concentrations between the LF and LFM formulations implies that their usage should be carefully calibrated based on specific soil needs and crop requirements.

**Fig. 2:**
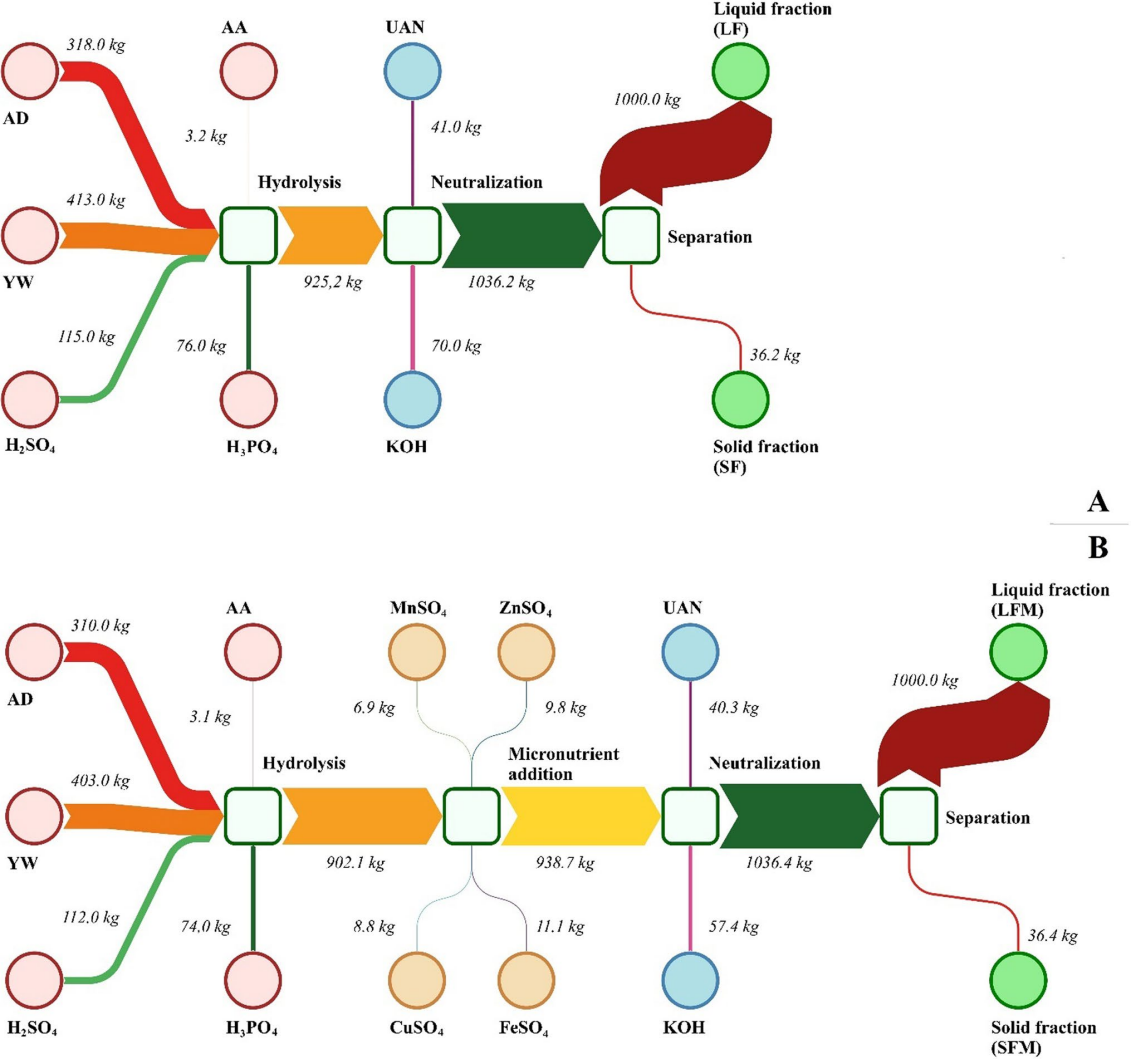
Material balance of applied preparations for LF (**A**) and LFM (**B**). Data are subject to 15% measurement uncertainty according to the ISO 17025 quality management system

Figure 2 shows the mass balances of all components used during fertilizer production. Their aim is to provide a visual representation of the process along with the raw materials required for its operation. According to previous studies, none of the macronutrients, especially nitrogen, is emitted during the process. This has been achieved through the use of an acidic pH, which makes it possible to reduce ammonia emissions (Wang et al. 2020a). The content of the solid fraction obtained after the separation process has also been shown to be less than 4% of the final product. This was achieved by liquefying as much as possible during the hydrolysis process (Germec et al. 2016; Lenihan et al. 2010). This allows most of the nutrients from the nutrients to be delivered to the finished liquid product. This type of process significantly reduces the need to manage the remaining solid fraction.

**Fig. 3:**
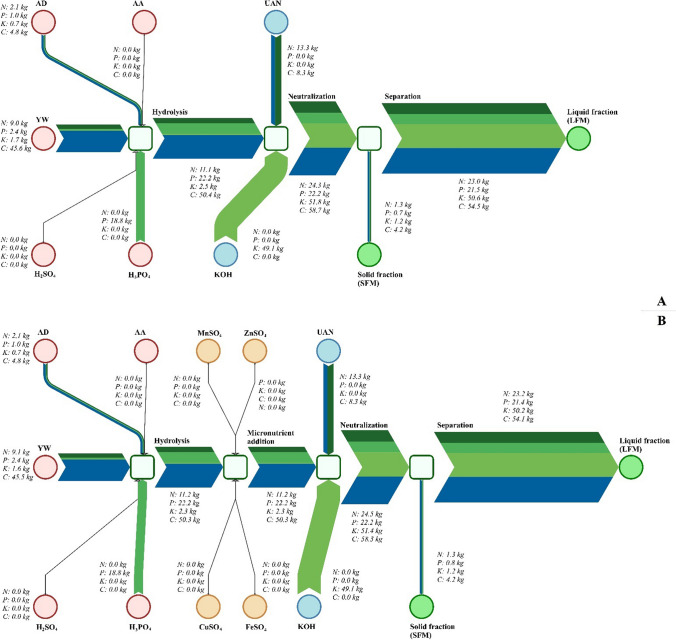
Nutrient balance of the prepared products applied for LF (**A**) and LFM (**B**). Data are subject to 15% measurement uncertainty according to the ISO 17025 quality management system

Figure 3 shows the streams of the most important fertilizer components like nitrogen, phosphorus, potassium and carbon at each stage of the process. The components of each of the raw materials used are listed. The design of this process guide allows for optimal adjustment of parameters in case of changes in the composition of raw materials. The most notable is the stream of macronutrients (N, P, K, C) for the solid fraction, which represents less than 5% of the total stream of macronutrients obtained in the process. This is advantageous due to the achievement of significant (>95%) efficiency of nutrient usage in the final fertilizer product.

#### Microbial analysis

Liquid fertilizers (LF and LFM) and raw materials used for their production (DS and YW) were characterized in terms of microbiological purity (Table 5). The total number of microorganisms (TNM) in DS determined on PCA medium was 3.4⋅10^5^ CFU/ml and was similar to the value obtained in the previous work (Skrzypczak et al. 2023), where DS received from the same manufacturer but from a different production batch was used. However, the current material was characterized by a lower density of fungi, including yeasts and filamentous fungi, than the previous batch. While no yeast but 40 CFU/ml of filamentous fungi were detected in the previous material, none of these groups of organisms was observed in the current samples. Similarly, the coliform number was almost two logarithmic orders lower. At the same time, the presence of *E. coli* and *Salmonella* sp. was not detected in any case. DS was produced exclusively on the basis of food wastes, which are usually not as rich a source of enteropathogens as municipal waste and human and animal manure (Manyi-Loh et al. 2016; Zhang et al. 2023). However, plant material can also be a source of this type of microorganisms due to the contact with soil (Patel et al. 2014). Yeast waste (YW) from brewing was characterized by a very low content of live microorganisms. TNM and coliforms, including *E. coli* and *Salmonella* sp., were not detected in the tested YW samples. However, a small number of live yeast cells were detected (≈200 CFU/ml).

**Table 5:** Microbiological parameters of liquid fertilizers (LF, LFM) and raw materials used for their production (DS, YW). Total number of microorganisms (TNM), total number of fungi including yeast and molds (TNF), not detected (ND)

Sample	TNM	TNF	Coliforms	*E. coli*	*Salmonella sp.* [in 25 g]
	*CFU/ml*	*CFU/ml*	*CFU/ml*	*CFU/ml*	
DS	3.7⋅10^5^	<10	1.1⋅10^3^ ± 2.1⋅10^1^	<10	ND
YW	<10	1.9⋅10^2^ ± 8.5⋅10^1^	<10	<10	ND
LF	1.5⋅10^3^ ± 7.1⋅10^2^	<10	<10	<10	ND
LFM	1.4⋅10^3^ ± 2.1⋅10^2^	<10	<10	<10	ND

The share of DS and YW in liquid fertilizer formulations was about 30% and 40% (w/v), respectively. These raw materials were therefore the source of bacteria and yeast in the liquid fertilizers, LF and LFM. DS introduced about 1⋅10^5^ CFU of TNM and 3⋅10^2^ CFU of coliforms (*E. coli* was not detected), while YW provided about 80 CFU of yeasts per 1 ml of liquid fertilizers. The process of sanitizing performed in LF and LFM with a mixture of sulfuric and phosphoric acids reduced TNM and the number of coliforms by two logarithmic orders. The groups of coliforms and yeasts were reduced to the undetectable levels by the methods used in this work. According to EU Regulation 2019/1009 of the European Parliament and of the Council (EUR-Lex 2023), the product can be used in agriculture as a mineral-organic fertilizer if the number of *E. coli* does not exceed 103 CFU per g or ml and *Salmonella* sp. is undetectable in 25 g or ml. Our results indicate that both LF and LFM meet the requirements of the standard.

#### Extraction tests

Extraction tests provide an opportunity to discern the forms in which nutrients are encapsulated in fertilizers. This knowledge proves crucial in assessing nutrient bioavailability to plants. High concentrations of nutrients do not necessarily equate to superior bioavailability, underscoring the importance of this factor in developing new fertilizer products. Nutrient bioavailability is the measure of a nutrient present on the substrate that the plant can uptake efficiently (Jones et al. 2013). Elements mainly accumulate at the surface of the soil, as most trace elements adsorb onto organic materials or form stable compounds with them (Saha et al. 2008). Nutrients in the soil are also present in the subsoil, including in the form of free ions, well-soluble or readily exchangeable forms, and ions bound to the organic matrix or solid phase (silicate minerals). Elements in the form of ions or their complexes have the highest bioavailability, but are very quickly leached into deeper soil layers. The soluble forms of nutrients are in the form of oxides and salts. When nutrients are deficient in the soil, deficiencies are made up by ion exchange. For forms that are difficult to solubilize, greater nutrient availability is achieved through additional treatments, such as chelation, changing the redox potential, or soil pH (Dean 2007). Plants also affect the availability of increased elements. Roots can release soluble organic compounds into the rhizosphere, which have the ability to complex micronutrients and thus potentially increase their uptake by plants (bioavailability) (Brun et al. 2001).

In this work, the content of water-soluble elements and the ammonium citrate-soluble form were determined. Studies indicate that the elemental composition of the fertilizer is inadequate to determine its bioavailability for plants (Degryse et al. 2020). The study included two types of extraction to fully demonstrate the potential of the fertilizer’s components. Analyses were performed in accordance with current PN-EN standards. The procedure dedicated to the identification of bioavailable forms of phosphorus (PN-EN 15958) was also adopted for the other components (K, Mg, S, Cu, Fe, Mn, and Zn) (Tables 6 and 7). One hundred percent solubility in both water and neutral ammonium citrate was recorded for SO_3_, which was derived from the sulfuric acid(VI) used in the hydrolysis process. An analogous effect was observed for MgO, whose sources were AD and YW, and K_2_O derived from potassium hydroxide was used during neutralization. In the case of phosphorus, the source of which was both waste and phosphoric acid(V) used for hydrolysis, there was approximately 20% less of the extracted component in water than in ammonium citrate for LF and approximately 15% less for LFM. The lack of total solubility of phosphorus in water may be due to the presence of hard-soluble forms in AD and YW. For micronutrients in the Cu, Mn, and Zn group, their solubility in water ranged from 50 to 100%, while in ammonium citrate, it ranged from 60 to 100%. The lowest solubility in both water and neutral ammonium citrate was recorded for iron. The component easily transforms in an alkaline environment into a form in the third oxidation state, which is not soluble in water and at the same time is not available to plants (Jones 2020). The lower content of water-soluble and ammonium citrate-soluble copper for LFM, derived mainly from sulfate salts, is due to precipitation of the component in the form of CuO (Kleinübing et al. 2010).

**Table 6:** Extraction tests of macronutrients in water (K_2_O, MgO, SO_3_ — PN-EN 15477; P_2_O_5_ — PN-EN 15958) and neutral ammonium citrate (PN-EN 15958) for LF and LFM. Data are subject to 15% measurement uncertainty according to the ISO 17025 quality management system

Sample	Medium	Release			
		%			
		K_2_O	P_2_O_5_	MgO	SO_3_
LF	*Water*	100	80.4	95.3	100
	*Ammonium citrate (pH 7)*	100	100	100	100
LFM	*Water*	100	87.1	100	100
	*Ammonium citrate (pH 7)*	100	97.8	100	100

**Table 7:** Micronutrient extraction tests in water (PN-EN 16962) and ammonium citrate (PN-EN 15958) for LF and LFM. Data are subject to 15% measurement uncertainty according to the ISO 17025 quality management system

Sample	Medium	Release			
		%			
		Cu	Fe	Mn	Zn
LF	*Water*	91.3	9.74	100	100
	*Ammonium citrate (pH 7)*	100	60.0	100	100
LFM	*Water*	52.3	65.8	85.6	95.8
	*Ammonium citrate (pH 7)*	69.1	77.2	100	95.8

### Plant tests

Utilizing waste from anaerobic digestion (AD) to fertilize crop fields can improve soil health improvement by increasing microbial activity and nutrient availability. Depending on the raw materials used in the AD process, the digestate may also contain contaminants, including toxic metals, pathogens, or even antibiotics. Therefore, the application of waste as fertilizer without prior treatment can be dangerous to the environment and future consumers (Vaish et al. 2022). In addition to the direct environmental hazards, using untreated digestate as fertilizer could negatively impact crop yields and the quality of the produce. The presence of contaminants like toxic metals, pathogens, and antibiotics can interfere with plant growth and development, leading to stunted crops or lower overall yields. These could be absorbed by the plants and accumulate within their tissues, consequently entering the food chain when the plant is consumed. This reduces the quality and safety of the product, but it also poses health risks to consumers, such as heavy metal poisoning or antibiotic resistance. Their residues in the soil can cause long-term degradation of soil fertility and affect future crop productivity. Processing the digestate to fertilizer is of paramount importance to preserve crop health, ensure produce quality, and safeguard both environmental and human health. The digestate can be subjected to strong mineral acids, which will improve hygienic quality and hydrolyze organic nitrogen into valuable amino acids (Skrzypczak et al. 2023). In this study, a mixture of sulfuric(VI) and phosphoric(V) acid was utilized to sanitize the digestate. In the methodology of our study, the sanitation and hydrolysis process of the digestate was carried out under carefully controlled conditions. Thirty test runs were conducted to ensure the consistency and reliability of the results. Each test was run with a specific ratio of the sulfuric(VI) and phosphoric(V) acid, ranging from a 1:5 to a 1:20 ratio by volume to digestate. The duration and medium concentrations of the acid treatment were also varied in a systematic way to identify the optimal conditions for sanitation and hydrolysis. The treated samples were then compared with the control samples to assess the efficiency of the acid treatment in sanitizing the digestate and hydrolyzing the organic nitrogen. To confirm the effectiveness of this process, liquid fertilizers (LF) based on conditioned digestate were examined in pot tests (Table 8).

**Table 8:** Results of lettuce pot tests — single application and fertigation

Preparation/dose (%)/Application type	Fresh mass (average weight of one lettuce) [g]	Chlorophyll content [mg/m^2^]	Root		
			Length [cm]	Surface area [cm^2^]	Diameter [mm]
W	0.38 ± 0.06	142 ± 23^a^	19.4 ± 1.0^a,b^	1.92 ± 0.27^a,b^	0.316 ± 0.038
RF/50/SA	2.91 ± 0.44	125 ± 26	131 ± 89^c,d^	15.7 ± 12.5^c,d^	0.356 ± 0.059
LF/50/SA	3.09 ± 0.46	80.3 ± 19.4^a,b^	38.1 ± 17.7	3.75 ± 1.99^e,f^	0.308 ± 0.022
LFM/50/SA	3.74 ± 0.56	129 ± 33	338 ± 55^a,c,e,f^	37.0 ± 5.9^a,c,e,g,h^	0.350 ± 0.017
RF/50/FE	5.45 ± 0.82	155 ± 12^b^	303 ± 16^b,d,g,h^	36.0 ± 1.8^b,d,f,i,j^	0.377 ± 0.002
LF/50/FE	3.32 ± 0.50	112 ± 10	85.9 ± 9.1^e,g^	9.04 ± 1.57^g,i^	0.334 ± 0.038
LFM/50/FE	4.02 ± 0.60	123 ± 19	42.0 ± 19.6^f,h^	4.48 ± 2.51^h,j^	0.325 ± 0.051
W	0.38 ± 0.06	142 ± 22	19.4 ± 1.0^a^	1.92 ± 0.27^a^	0.316 ± 0.038
RF/100/SA	4.97 ± 0.75	97.7 ± 17.0^a^	75.4 ± 56.3^b^	8.79 ± 7.30^b^	0.338 ± 0.067
LF/100/SA	4.21 ± 0.63	111 ± 16	75.3 ± 11.1^c^	7.87 ± 1.14^c^	0.333 ± 0.020
LFM/100/SA	5.72 ± 0.86	120 ± 30	154 ± 84^d^	16.4 ± 10.1^d^	0.320 ± 0.050
RF/100/FE	7.89 ± 1.18	175 ± 10^a^	361 ± 125^a,b,c,d,e,f^	42.8 ± 13.3^a,b,c,d,e,f^	0.383 ± 0.033
LF/100/FE	2.61 ± 0.39	146 ± 38	30.2 ± 13.7^e^	3.81 ± 1.90^e^	0.398 ± 0.044
LFM/100/FE	6.39 ± 0.96	131 ± 19	92.4 ± 21.6^f^	10.2 ± 2.5^f^	0.351 ± 0.008
W	0.38 ± 0.06	142 ± 22^a^	19.4 ± 1.0^a,b^	1.92 ± 0.27	0.316 ± 0.038
RF/150/SA	2.49 ± 0.37	61.3 ± 9.7^a,b,c,d,e,f^	13.4 ± 6.7^c,d^	1.29 ± 0.92^A^	0.286 ± 0.060
LF/150/SA	3.96 ± 0.59	114 ± 16^b,g^	41.4 ± 13.2^e^	4.28 ± 1.48	0.328 ± 0.031
LFM/150/SA	4.71 ± 0.71	144 ± 13c	93.3 ± 14.7^a,c^	9.52 ± 0.76	0.329 ± 0.049
RF/150/FE	9.96 ± 1.49	173 ± 17^d,g,h^	129 ± 25^b,d,e,f,g^	18.9 ± 8.1^A^	0.455 ± 0.116
LF/150/FE	3.32 ± 0.50	116 ± 12^e,h^	38.8 ± 26.4^f^	4.09 ± 2.55	0.341 ± 0.044
LFM/150/FE	5.95 ± 0.89	128 ± 28^f^	57.0 ± 33.5^g^	6.07 ± 3.62	0.332 ± 0.044

^a,b,c^The results marked with the same letter differ statistically significant — Tukey’s test (RIR) test (*p* < 0.05, vertical comparison with the same dose of fertilizer and control group)

^A,B,C^Results marked with the same letter differ statistically significantly — Kruskal-Wallis test (*p* < 0.05, vertical comparison with the same dose of fertilizer and control group)

Based on the results of the lettuce pot test displayed in Table 8, several important conclusions can be drawn about the influence of different fertilizer preparations and application methods on lettuce growth parameters. For instance, the control group, denoted as “W,” had the lowest values for fresh mass and root parameters such as length and surface area. This suggests that all fertilizer preparations and application methods improved lettuce growth compared to the control condition.

Different fertilizers and application methods, namely single application (SA) and fertigation (FE), were compared at 50%, 100%, and 150% doses. For most fertilizers, fertigation generally led to a higher fresh mass of lettuce compared to a single application. The group “RF/50/FE” had a notable increase in fresh mass, achieving 5.45 g compared to 2.91 g observed in the “RF/50/SA” group. The chlorophyll content did not exhibit a clear trend across the various fertilizer preparations and application methods, suggesting that these factors may not be the dominant influences on chlorophyll concentration in the tested lettuce plants.

The root parameters, encompassing length, surface area, and diameter, were typically superior with fertigation at 50% dose, compared to single application. The “RF/50/FE” and “LFM/50/FE” groups showed particularly significant root lengths and surface areas. The increase of the fertilizer dose from 50 to 100% and 150% generally improved the fresh mass for the “RF” type of fertilizer when applied through fertigation, although this pattern was not consistent for the “LF” and “LFM” fertilizer types. Statistical analysis revealed significant differences among the tested groups, contributing to a more nuanced understanding of the influences of various fertilizers and application methods on lettuce growth. This study indicates that both the type of fertilizer and its method of application have a significant impact on the growth parameters of lettuce plants, with fertigation generally producing superior outcomes compared to single application. Future research could further investigate the long-term impacts and potential environmental effects of these various fertilizer strategies.

The research did not reveal a phytotoxic effect. All lettuces treated with LF had much higher biometric parameters than the control group (unfertilized, watered only), for example, the LF/50/SA group had about eight times higher yield and almost two times the root length. AD by-products of AD are mainly a source of NPK, but can be converted into full-quality fertilizers by adding micronutrients (Robles-Aguilar et al. 2019). Despite the lower demand for microelements, they are essential to maintain proper plant development and growth. They perform many important functions such as chlorophyll synthesis, enzyme activation, and molecular nitrogen fixation (Tripathi et al. 2015). Therefore, liquid LFM fertilizers containing micronutrients such as Cu, Mn, Zn, and Fe were also used for pot tests. Several benefits of introducing micronutrients into fertilizers have been documented in agricultural research. For instance, copper (Cu) plays a critical role in plant resistance to diseases and improves the quality of fruits and grains (Marschner 2011). Similarly, manganese (Mn) has been found to be vital in photosynthesis and nitrogen metabolism, with deficiencies leading to reduced crop yields (Broadley et al. 2012). Zinc (Zn) is instrumental in DNA synthesis, protein production, and growth regulation, hence impacting crop productivity and nutritional quality (Cakmak 2008). Moreover, iron (Fe) is fundamental for chlorophyll synthesis, and its deficiency can result in chlorosis, impairing photosynthetic efficiency and crop productivity (Morrissey and Guerinot 2009). The biometric parameters of lettuce prove that correction of fertilizer composition by the introduction of micronutrients (LFM) can bring even more benefits: higher chlorophyll content, longer roots, and higher yields than in groups without micronutrients (LF). Therefore, the valorization of digestate may be a promising solution for agriculture, especially in these times of high demand for fertilizers (Keskinen et al. 2020).

Fertigation is a relatively new method in agriculture that allows for a more efficient distribution of water and fertilizer through special irrigation systems (Ranjan and Sow 2021). Fertigation, a combination of fertilization and irrigation, delivers nutrients and water directly to the plant roots through an irrigation system. This approach ensures that nutrients are applied precisely when and where the plants need them, thereby minimizing waste and maximizing uptake efficiency. It offers significant advantages over traditional surface broadcast application methods, which can result in nutrient losses due to runoff, leaching, or evaporation. Fertigation is beneficial where water availability is limited, in arid or semi-arid regions. Delivering water and nutrients directly to the root zone causes minimization of water use and ensures that the plant makes the most efficient use of the nutrients. This system also reduces the amount of labor and machinery needed for nutrient application, providing added cost and energy savings. Fertigation allows for flexibility in nutrient management. For instance, nutrient applications can be adjusted throughout the growth season based on plant needs, thus potentially improving crop yields and quality. This method is also beneficial in scenarios with high-value crops like fruits, vegetables, or ornamentals, where optimal nutrient management is essential for maximizing economic returns. In plant trials, we observed the impact of digestion application methods — soil application and fertigation — on lettuce yields. These trials aimed to determine the most effective application method for our organic-mineral fertilizers and to investigate how different application strategies influence crop performance. The findings of these trials will provide valuable insights into how best to use these fertilizers for optimal plant growth and productivity The collected results show that regardless of the applied rate (50, 100, or 150% of nitrogen requirements), the fertigation with micronutrients resulted in the higher yields than in groups where traditional fertilization was applied (from 7 to even 20% more). However, the opposite relationship was observed for root ball parameters, such as length and surface area. In the groups where LFM fertilizer was applied to the soil, lettuces exhibited roots up to eight times longer than those where fertigation was applied at a dose of 50% (statistically significant results, *p* < 0.05). Fertigation helps maintain optimal soil moisture. Svobodová et al. (2023) conducted a field study of potatoes where they applied pre-sown fertilization and fertigation. The yield in the irrigated group was approximately 40% higher than in the nonirrigated group (Svobodová et al. 2023). The positive effects of applying the digestate as a biofertilizer supplied to tomatoes using a drip fertigation system have also been reported by Barzee et al. (2019). They used two types of digestate, derived from dairy manure or food scraps. The first residue gave a higher yield (more than 7 tons/ha) than the second (6.26 tons/ha). Despite promising results, scientists have encouraged long-term studies that will provide a better view of the environmental impact, particularly on the soil (Barzee et al. 2019). Long-term studies are imperative to holistically understand the implications of digestate use as a biofertilizer, assessing both its environmental impact and possible enduring effects on crop yields. These investigations ought to concentrate on various crucial parameters.

The nitrogen content in plants fertilized with LF and LFM through fertigation was invariably higher than in plants fertilized with the same dose of reference fertilizer (Table 9). In single applications, the differences were relatively minor, suggesting that nitrogen might be lost during cultivation, limiting the plant’s ability to assimilate it (Jia et al. 2014). This observation aligns with the physiological test results, as each test group demonstrated significantly lower chlorophyll content for the reference fertilizer compared to the test fertilizers is consistent with the results of many publications showing a correlation between nitrogen content in the plant and photosynthetic intensity (Kovács and Vyn 2017; Peng et al. 2016; Smith et al. 2022). Although there are two times differences in tissues (6.30% for LF/50/SA vs. 2.69% RF/50/SA), the change in soil nitrogen content is not noticeable and in all cases is within the range of 0.443 to 0.945%. On the contrary, the tissue K content is lower for all cases for fertigation applications. In soil fertilized at a rate of 150% for all fertilizers tested, the potassium content is higher than in the other rates. However, this is not reflected in the potassium content of the plant, which may indicate that the environment is saturated with this element beyond its assimilation potential (Zalewska et al. 2018). The manganese content in tissues for doses of 100% and 150% is in each case higher for LFM fertilizer then for LF and RF. Since a lack of adequate micronutrient supply can be a limiting factor, supplementation is essential for high yielding cultivations (Zulfiqar et al. 2021). Additionally, lettuce leaves can be biofortified with this micronutrient. Due to its role in metabolism, especially as an antioxidant, such biofortified food can be a valuable nutrient (Chen et al. 2018).

**Table 9:** Multielemental composition of lettuce and soil from pot tests

Samples	Macroelements				Microelements			
	N	C	P	K	Cu	Fe	Mn	Zn
	*%*	*%*	*%*	*%*	*mg/kg*	*mg/kg*	*mg/kg*	*mg/kg*
W	4.81 ± 0.48	40.8 ± 4.1	0.116 ± 0.023	3.45 ± 0.69	18.2 ± 2.7	261 ± 39	106 ± 16	153 ± 23
RF/50/SA	2.69 ± 0.27	39.6 ± 4.0	0.509 ± 0.102	4.73 ± 0.95	3.07 ± 0.46	136 ± 20	40.5 ± 6.1	65.5 ± 9.8
RF/50/FE	3.52 ± 0.35	41.8 ± 4.2	0.332 ± 0.066	2.51 ± 0.50	0.761 ± 0.114	153 ± 23	23.4 ± 3.5	32.0 ± 4.8
LF/50/SA	6.30 ± 0.63	36.3 ± 3.6	0.852 ± 0.170	6.31 ± 1.26	1.24 ± 0.19	159 ± 24	77.4 ± 11.6	69.3 ± 10.4
LF/50/FE	5.22 ± 0.52	38.0 ± 3.8	0.749 ± 0.150	5.47 ± 1.09	38.8 ± 5.8	162 ± 24	95.7 ± 14.4	75.0 ± 11.3
LFM/50/SA	3.81 ± 0.38	38.4 ± 3.8	0.723 ± 0.145	5.61 ± 1.12	41.1 ± 6.2	121 ± 18	142 ± 21	77.2 ± 11.6
LFM/50/FE	5.14 ± 0.51	37.6 ± 3.8	0.857 ± 0.171	6.30 ± 1.26	25.6 ± 3.8	190 ± 28	143 ± 21	164 ± 25
RF/100/SA	5.44 ± 0.54	37.1 ± 3.7	0.727 ± 0145	5.72 ± 1.14	13.9 ± 2.1	150 ± 23	39.3 ± 5.9	75.3 ± 11.3
RF/100/FE	3.85 ± 0.38	40.8 ± 4.1	0.437 ± 0.087	2.98 ± 0.60	2.54 ± 0.38	180 ± 27	33.4 ± 5.0	38.5 ± 5.8
LF/100/SA	6.10 ± 0.61	36.2 ± 3.6	0.987 ± 0.197	6.21 ± 1.24	0.487 ± 0.073	173 ± 26	97.5 ± 14.6	49.2 ± 7.4
LF/100/FE	6.30 ± 0.63	38.2 ± 3.8	0.770 ± 0.154	5.37 ± 1.07	10.8 ± 1.6	166 ± 25	101 ± 15	76.7 ± 11.5
LFM/100/SA	5.68 ± 0.57	37.4 ± 3.7	0.899 ± 0.180	6.31 ± 1.26	17.8 ± 2.7	211 ± 32	249 ± 37	108 ± 16
LFM/100/FE	5.42 ± 0.54	36.3 ± 3.6	1.00 ± 0.20	6.51 ± 1.30	48.3 ± 7.2	142 ± 21	213 ± 32	141 ± 21
RF/150/SA	7.16 ± 0.72	31.7 ± 3.2	0.876 ± 0.175	7.16 ± 1.43	<LOD	170 ± 25	35.9 ± 5.4	59.6 ± 8.9
RF/150/FE	5.60 ± 0.56	38.6 ± 3.9	0.561 ± 0.112	4.13 ± 0.83	<LOD	109 ± 16	24.4 ± 3.7	29.2 ± 4.4
LF/150/SA	6.96 ± 0.70	34.8 ± 3.5	0.924 ± 0.185	5.88 ± 1.18	<LOD	138 ± 21	108 ± 16	58.2 ± 8.7
LF/150/FE	7.10 ± 0.71	35.0 ± 3.5	0.817 ± 0.163	5.74 ± 1.15	8.81 ± 1.32	143 ± 21	139 ± 21	66.9 ± 10.0
LFM/150/SA	7.31 ± 0.73	36.9 ± 3.7	0.880 ± 0.176	5.94 ± 1.19	38.2 ± 5.7	154 ± 23	192 ± 29	95.3 ± 14.3
LFM/150/FE	7.35 ± 0.73	35.7 ± 3.6	0.887 ± 0.177	6.17 ± 1.23	37.0 ± 5.5	107 ± 16	169 ± 25	130 ± 19
S/W	0.683 ± 0.068	18.3 ± 1.8	0.0105 ± 0.0021	0.0490 ± 0.0073	<LOD	1019 ± 204	11.3 ± 1.7	5.15 ± 0.8
S/RF/50/SA	0.635 ± 0.063	19.5 ± 1.9	0.0115 ± 0.0023	0.0653 ± 0.0098	<LOD	1252 ± 250	17.2 ± 2.6	3.05 ± 0.5
S/RF/50/FE	0.745 ± 0.074	20.9 ± 2.1	0.0182 ± 0.0036	0.0544 ± 0.0082	<LOD	1218 ± 244	22.3 ± 3.3	34.4 ± 5.2
S/LF/50/SA	0.796 ± 0.080	22.8 ± 2.3	0.0141 ± 0.0028	0.0721 ± 0.0108	<LOD	1067 ± 213	14.3 ± 2.2	18.5 ± 2.8
S/LF/50/FE	0.636 ± 0.064	16.5 ± 1.6	0.0204 ± 0.0041	0.0526 ± 0.0079	<LOD	1248 ± 250	21.7 ± 3.3	4.43 ± 0.7
S/LFM/50/SA	0.660 ± 0.066	18.5 ± 1.8	0.0189 ± 0.0038	0.0633 ± 0.0095	<LOD	1281 ± 256	21.7 ± 3.3	4.17 ± 0.6
S/LFM/50/FE	0.916 ± 0.092	25.6 ± 2.6	0.0155 ± 0.0031	0.0643 ± 0.0097	17.7 ± 2.6	987 ± 148	41.5 ± 6.2	24.4 ± 3.7
S/RF/100/SA	0.443 ± 0.044	13.7 ± 1.4	0.0155 ± 0.0031	0.0526 ± 0.0079	<LOD	966 ± 145	12.7 ± 1.9	5.27 ± 0.8
S/RF/100/FE	0.880 ± 0.088	24.8 ± 2.5	0.0136 ± 0.0027	0.0471 ± 0.0071	<LOD	1007 ± 201	19.9 ± 3.0	7.97 ± 1.2
S/LF/100/SA	0.808 ± 0.081	22.6 ± 2.3	0.0140 ± 0.0028	0.0508 ± 0.0076	<LOD	731 ± 110	8.59 ± 1.3	3.37 ± 0.5
S/LF/100/FE	0.711 ± 0.071	18.7 ± 1.9	0.0392 ± 0.0078	0.0908 ± 0.0136	<LOD	1526 ± 305	24.6 ± 3.7	6.07 ± 0.9
S/LFM/100/SA	0.706 ± 0.071	19.3 ± 1.9	0.0235 ± 0.0047	0.0902 ± 0.0135	21.3 ± 3.2	1270 ± 254	48.7 ± 7.3	27.7 ± 4.2
S/LFM/100/FE	0.692 ± 0.070	18.7 ± 1.9	0.0255 ± 0.0051	0.0737 ± 0.0111	39.5 ± 5.9	1128 ± 226	57.7 ± 8.7	37.6 ± 5.6
S/RF/150/SA	0.614 ± 0.061	18.6 ± 1.9	0.0173 ± 0.0035	0.0603 ± 0.0090	<LOD	1040 ± 208	14.2 ± 2.1	6.00 ± 0.9
S/RF/150/FE	0.842 ± 0.084	21.2 ± 2.1	0.0181 ± 0.0036	0.0555 ± 0.0083	<LOD	1430 ± 286	25.0 ± 3.8	8.16 ± 1.2
S/LF/150/SA	0.832 ± 0.083	23.1 ± 2.3	0.0295 ± 0.0059	0.120 ± 0.024	<LOD	1178 ± 236	40.1 ± 6.0	141 ± 21
S/LF/150/FE	0.945 ± 0.094	23.6 ± 2.4	0.0373 ± 0.0075	0.127 ± 0.025	<LOD	1438 ± 288	31.0 ± 4.6	13.1 ± 2.0
S/LFM/150/SA	0.740 ± 0.074	19.5 ± 1.9	0.0287 ± 0.0057	0.107 ± 0.021	40.7 ± 6.1	1138 ± 228	59.2 ± 8.9	58.2 ± 8.7
S/LFM/150/FE	0.631 ± 0.063	16.5 ± 1.6	0.0304 ± 0.0061	0.111 ± 0.022	40.2 ± 6.0	1331 ± 266	65.5 ± 9.8	46.0 ± 6.9

The enduring influence of digestate on soil health is one of the key considerations. It necessitates a meticulous examination of alterations in soil composition, fertility, organic matter quantity, and microbial vitality over a substantial period. While digestate can enhance soil health in the short term by supplying essential nutrients, further studies are needed to ascertain the consequences on soil fertility in the long run.

These investigations must scrutinize the lasting impact on crop yields and quality. It is encouraging to see immediate yield enhancements, but we need to understand if these improvements can be sustained over multiple growing seasons. Another significant factor is the potential effect on groundwater and surface water quality. This involves studying nutrient leaching and runoff, specifically nitrogen and phosphorus, which are known contributors to water pollution. Also, the influence on greenhouse gas emissions cannot be overlooked. The application of digestate could potentially minimize the demand for synthetic fertilizers. This results in lower CO_2_ emission. Organic fertilizers can contribute to the emission of nitrous oxide, a powerful greenhouse gas.

## Future perspective

Waste is a valuable source of raw material for biogas plants. During the production of biofuel, a large amount of waste is generated in the form of digestate, which requires further management. Due to its high proportion of nutrients, the waste stream can be reused for fertilizer production. The search for renewable raw materials in the current fertilizer crisis is particularly important. Direct application of the digestate to cultivated soils poses serious risks to the environment and consumers (spread of pathogenic microorganisms, emission of greenhouse gases, adverse change of soil microflora). One solution is to perform acid treatment, which, in addition to sanitization, allows conversion of organic nitrogen to short peptides and amino acids (a stimulating effect on plants).

The proposed technology of full-strength fertilizers based on digestate and yeast (a source of nitrogen and organic carbon) was brought to technology level 5 (TRL 5). The effectiveness of the product has been confirmed in an environment that simulates real conditions (pot tests, soil application/fertilization). However, the implementation of the proposed technology requires a number of additional steps including process scaling, verification of process parameters at the technical scale, and checking the stability of raw materials and produced formulations (stability of composition and microbiological purity). A key study in this regard is also to conduct field tests that will confirm the effectiveness and safety of the fertilizer under long-term real-world conditions over the full growing season (advantageously by a certification body).

The presented results offer a promising solution for fertilizer companies to become less dependent on dwindling mineral resources. Technologies based on renewable resources are the future of sustainable agriculture. Cooperation between researchers and fertilizer manufacturers is key, bringing sustainability closer.

## Conclusion

The utilization of biogas plant digestate and brewery yeast waste for the creation of fertilizers demonstrates significant promise. The optimized acid hydrolysis process facilitated the extraction of key plant nutrients, such as nitrogen, carbon, potassium, phosphorus, and magnesium, as well as valuable biostimulatory free amino acids. These fertilizers, meeting the European Parliament Regulation 2019/1009 (EUR-Lex 2023) and plants nutritional requirements, present a sustainable alternative to traditional, non-renewable options. Waste treatment with a mixture of sulfuric(VI) and phosphoric(V) acid served a dual purpose, providing both material sanitization and an increase in free amino acids. The developed fertilizers exhibited high nutrient bioavailability, confirmed through extraction tests. In our pot tests with lettuce, the method of fertilizer application — whether single application or fertigation — noticeably affected plant growth parameters and yield. In particular, fertigation with the micronutrient formulation resulted in a marked increase in fresh plant mass. Simultaneously, these fertilizers enabled significant biofortification of plant biomass in both macro- and micronutrients. This innovative approach of utilizing treated waste as fertilizer resources can lead to a reduction in non-renewable raw material consumption and mitigate environmental impact, particularly concerning greenhouse gas emissions.

### Supplementary information

ESM 1 (DOCX 30 kb)

## Data Availability

The data and materials can be available on request.

## Abbreviations

1H100: Primary fertilizer formulation hydrolyzed for 1 h with 100% acid solution
1H40: Primary fertilizer formulation hydrolyzed for 1 h with 40% acid solution
1H60: Primary fertilizer formulation hydrolyzed for 1 h with 60% acid solution
24H100: Primary fertilizer formulation hydrolyzed for 24 h with 100% acid solution
24H40: Primary fertilizer formulation hydrolyzed for 24 h with 40% acid solution
24H60: Primary fertilizer formulation hydrolyzed for 24 h with 60% acid solution
6H100: Primary fertilizer formulation hydrolyzed for 6 h with 100% acid solution
6H40: Primary fertilizer formulation hydrolyzed for 6 h with 40% acid solution
6H60: Primary fertilizer formulation hydrolyzed for 6 h with 60% acid solution
AA: Antifoaming agent
AD: Anaerobic digestion
CTS: Catalytic solution
DS: Anaerobic digestate
DWM: Diluting and washing medium
ELM: Eluting medium
ESI: Electrospray ionization mass spectrometry
EQS: Sorbent equilibration medium
FE: Fertigation application
H1H100: Hydrolysate produced after 1 h with the addition of 100% acid solution without neutralization
H1H40: Hydrolysate produced after 1 h with the addition of 40% acid solution without neutralization
H1H60: Hydrolysate produced after 1 h with the addition of 60% acid solution without neutralization
H24H100: Hydrolysate produced after 6 h with the addition of 100% acid solution without neutralization
H24H40: Hydrolysate produced after 6 h with the addition of 40% acid solution without neutralization
H24H60: Hydrolysate produced after 6 h with the addition of 60% acid solution without neutralization
H6H100: Hydrolysate produced after 6 h with the addition of 100% acid solution without neutralization
H6H40: Hydrolysate produced after 6 h with the addition of 40% acid solution without neutralization
H6H60: Hydrolysate produced after 6 h with the addition of 60% acid solution without neutralization
ICP-OES: Inductively coupled plasma-optical emission spectrometry
IS: Internal standard
LF: Liquid fertilizer without micronutrients
LFM: Liquid fertilizer with micronutrients
MSPE: Microfilter solid-phase extraction
PM: Precipitation medium
RDS: Reagent solution
RF: Reference fertilizer
SA: Single application
S1H100: Solid fraction formed after hydrolysis for 1 h with 100% acid solution
S1H40: Solid fraction formed after hydrolysis for 1 h with 40% acid solution
S1H60: Solid fraction formed after hydrolysis for 1 h with 60% acid solution
S24H100: Solid fraction formed after hydrolysis for 24 h with 100% acid solution
S24H40: Solid fraction formed after hydrolysis for 24 h with 40% acid solution
S24H60: Solid fraction formed after hydrolysis for 24 h with 60% acid solution
S6H100: Solid fraction formed after hydrolysis for 24 h with 100% acid solution
S6H40: Solid fraction formed after hydrolysis for 6 h with 40% acid solution
S6H60: Solid fraction formed after hydrolysis for 6 h with 60% acid solution
TNF: Total number of fungi
TNM: Total number of microorganisms
W: Water
WES: Sorbent activation medium
YW: Yeast waste

## Symbols

*C*_E(i)_: Element concentration in contact solution (mg/l)
*C*_m(i)_: Element concentration in extracted materials (mg/kg)
*m*_m_: Mass of extracted materials (kg)
*V*: Extract volume (ml)
*C*_F(i)_: Form in fertilizer (%)
