# Supplementary material for: Recent innovations in fertilization with treated digestate from food waste to recover nutrients for arid agricultural fields
Source: Environ Sci Pollut Res Int. 2023 Dec 5;31(29):41563–85. doi: 10.1007/s11356-023-31211-2 (PMC11219367; doi:10.1007/s11356-023-31211-2)
Supplement: Supplementary file 1 — (DOCX 30 kb) [file 11356_2023_31211_MOESM1_ESM.docx]

**Supplementary materials**

**Table S1.** *Analysis of selected physicochemical properties of raw materials*

| Materials | Properties | | | |
| --- | --- | --- | --- | --- |
|  | **Viscosity** | **Dry mass** | **Density** | **pH** |
|  | *mPa⋅s* | *%* | *kg/m^3^* |  |
| DS | 82.1 | 37.0 | 1016 | 8.0 |
| YW | 3894 | 21.6 | 1094 | 6.3 |

**Table S2.** *Multielemental analysis of preparation, hydrolysates, and solid fraction from optimalization process*

| Samples | Macroelements | | | | | | | | Microelements | | | | |
| --- | --- | --- | --- | --- | --- | --- | --- | --- | --- | --- | --- | --- | --- |
|  | **C** | **N** | **P_2_O_5_** | **K_2_O** | **CaO** | **MgO** | **Na_2_O** | **SO_3_** | **Cu** | **Fe** | **Mn** | **Zn** |  |
|  | *%* | | | | | | | | *mg/L* | | | | |
| 1H40 | 3.85  ±0.39 | 2.17  ±0.22 | 2.02  ±0.30 | 3.77  ±0.57 | 0.0240  ±0.0036 | 0.0340  ±0.0051 | 0.181  ±0.027 | 2.86  ±0.43 | 10.3  ±1.5 | 77.4  ±11.6 | 5.35  ±0.80 | 3.09  ±0.46 |  |
| 1H60 | 3.63  ±0.36 | 2.17  ±0.22 | 3.06  ±0.46 | 4.45  ±0.67 | 0.0200  ±0.0030 | 0.0351  ±0.0053 | 0.194  ±0.029 | 2.66  ±0.40 | 6.93  ±1.04 | 48.4  ±7.3 | 4.27  ±0.64 | 3.27  ±0.49 |  |
| 1H100 | 3.42  ±0.34 | 2.38  ±0.24 | 5.38  ±0.81 | 5.55  ±0.83 | 0.0360  ±0.0054 | 0.0375  ±0.0056 | 0.234  ±0.035 | 2.12  ±0.32 | 1.91  ±0.29 | 30.5  ±4.6 | 4.26  ±0.64 | 4.57  ±0.69 |  |
| 6H40 | 3.78  ±0.38 | 2.20  ±0.22 | 2.11  ±0.32 | 3.65  ±0.55 | 0.0310  ±0.0047 | 0.0350  ±0.0053 | 0.183  ±0.027 | 2.74  ±0.41 | 2.77  ±0.42 | 33.1  ±5.0 | 4.04  ±0.61 | 2.77  ±0.42 |  |
| 6H60 | 3.61  ±0.36 | 2.19  ±0.22 | 3.14  ±0.47 | 4.36  ±0.65 | 0.0150  ±0.0023 | 0.0365  ±0.0055 | 0.202  ±0.030 | 2.80  ±0.42 | 2.03  ±0.30 | 50.0  ±7.5 | 4.75  ±0.71 | 3.80  ±0.57 |  |
| 6H100 | 3.66  ±0.37 | 2.40  ±0.24 | 5.20  ±0.78 | 6.05  ±0.91 | 0.0360  ±0.0054 | 0.0356  ±0.0053 | 0.228  ±0.034 | 2.43  ±0.36 | 2.89  ±0.43 | 39.4  ±5.9 | 4.31  ±0.65 | 5.61  ±0.84 |  |
| 24H40 | 3.78  ±0.38 | 2.21  ±0.22 | 2.11  ±0.32 | 4.10  ±0.62 | 0.0520  ±0.0078 | 0.0350  ±0.0053 | 0.182  ±0.027 | 3.20  ±0.48 | 3.57  ±0.54 | 31.8  ±4.8 | 3.32  ±0.50 | 1.92  ±0.29 |  |
| 24H60 | 3.59  ±0.36 | 2.13  ±0.21 | 3.21  ±0.48 | 4.51  ±0.68 | 0.0160  ±0.0024 | 0.0369  ±0.0055 | 0.206  ±0.031 | 2.97  ±0.45 | 6.07  ±0.91 | 37.1  ±5.6 | 5.29  ±0.79 | 3.03  ±0.45 |  |
| 24H100 | 3.50  ±0.35 | 2.34  ±0.23 | 5.30  ±0.80 | 6.45  ±0.97 | 0.0300  ±0.0045 | 0.0372  ±0.0056 | 0.242  ±0.036 | 3.22  ±0.48 | 3.86  ±0.58 | 61.8  ±9.3 | 4.89  ±0.73 | 5.54  ±0.83 |  |
| H1H40 | 5.61  ±0.56 | 2.45  ±0.25 | 2.01  ±0.30 | 3.78  ±0.57 | 0.130  ±0.020 | 0.0335  ±0.0050 | 0.160  ±0.024 | 3.16  ±0.47 | 2.71  ±0.41 | 170  ±26 | 5.88  ±0.88 | 6.18  ±0.93 |  |
| H1H60 | 5.27  ±0.53 | 2.28  ±0.23 | 2.78  ±0.42 | 6.72  ±1.01 | 0.151  ±0.023 | 0.0333  ±0.0050 | 0.168  ±0.025 | 5.20  ±0.78 | 3.25  ±0.49 | 173  ±26 | 6.02  ±0.90 | 6.74  ±1.01 |  |
| H1H100 | 4.53  ±0.45 | 2.18  ±0.22 | 4.53  ±0.68 | 10.6  ±1.6 | 0.133  ±0.020 | 0.0331  ±0.0050 | 0.191  ±0.029 | 9.47  ±1.42 | 3.24  ±0.49 | 169  ±25 | 6.12  ±0.92 | 7.94  ±1.19 |  |
| H6H40 | 5.64  ±0.56 | 2.35  ±0.24 | 2.03  ±0.30 | 4.06  ±0.61 | 0.182  ±0.027 | 0.0347  ±0.0052 | 0.164  ±0.025 | 3.38  ±0.51 | 3.17  ±0.48 | 178  ±27 | 5.83  ±0.87 | 6.25  ±0.94 |  |
| H6H60 | 5.33  ±0.53 | 2.27  ±0.23 | 2.88  ±0.43 | 6.52  ±0.98 | 0.153  ±0.023 | 0.0339  ±0.0051 | 0.169  ±0.025 | 5.42  ±0.81 | 3.18  ±0.48 | 190  ±29 | 5.86  ±0.88 | 6.89  ±1.03 |  |
| H6H100 | 4.57  ±0.46 | 2.22  ±0.22 | 4.43  ±0.66 | 10.6  ±1.6 | 0.178  ±0.027 | 0.0335  ±0.0050 | 0.191  ±0.029 | 9.11  ±1.37 | 2.93  ±0.44 | 170  ±26 | 5.44  ±0.82 | 6.41  ±0.96 |  |
| H24H40 | 5.53  ±0.55 | 2.28  ±0.23 | 2.03  ±0.30 | 4.51  ±0.68 | 0.185  ±0.028 | 0.0345  ±0.0052 | 0.164  ±0.025 | 3.83  ±0.57 | 3.03  ±0.45 | 180  ±27 | 6.18  ±0.93 | 7.51  ±1.13 |  |
| H24H60 | 5.43  ±0.54 | 2.30  ±0.23 | 2.86  ±0.43 | 7.04  ±1.06 | 0.166  ±0.025 | 0.0336  ±0.0050 | 0.172  ±0.026 | 5.92  ±0.89 | 3.72  ±0.56 | 192  ±29 | 5.81  ±0.87 | 7.00  ±1.05 |  |
| H24H100 | 4.54  ±0.45 | 2.23  ±0.22 | 4.57  ±0.69 | 10.4  ±1.6 | 0.165  ±0.025 | 0.0332  ±0.0050 | 0.195  ±0.029 | 9.20  ±1.38 | 3.55  ±0.53 | 164  ±25 | 5.92  ±0.89 | 8.03  ±1.20 |  |
| S1H40 | 7.52  ±0.75 | 2.53  ±0.25 | 1.96  ±0.29 | 4.21  ±0.63 | 0.207  ±0.031 | 0.0330  ±0.0050 | 0.158  ±0.024 | 3.64  ±0.55 | 7.02  ±1.05 | 276  ±41 | 6.10  ±0.92 | 10.6  ±1.6 |  |
| S1H60 | 6.56  ±0.66 | 2.25  ±0.23 | 2.62  ±0.39 | 7.08  ±1.06 | 0.183  ±0.027 | 0.0316  ±0.0047 | 0.158  ±0.024 | 5.74  ±0.86 | 5.22  ±0.78 | 270  ±41 | 7.38  ±1.11 | 9.27  ±1.39 |  |
| S1H100 | 5.38  ±0.54 | 2.04  ±0.20 | 3.97  ±0.60 | 13.6  ±2.0 | 0.190  ±0.029 | 0.0297  ±0.0045 | 0.169  ±0.025 | 13.1  ±2.0 | 4.57  ±0.69 | 258  ±39 | 6.64  ±1.00 | 9.07  ±1.36 |  |
| S6H40 | 6.76  ±0.68 | 2.33  ±0.23 | 1.95  ±0.29 | 4.42  ±0.66 | 0.185  ±0.028 | 0.0331  ±0.0050 | 0.156  ±0.023 | 3.69  ±0.55 | 5.40  ±0.81 | 295  ±44 | 7.67  ±1.15 | 10.3  ±1.5 |  |
| S6H60 | 6.68  ±0.67 | 2.20  ±0.22 | 2.64  ±0.40 | 6.94  ±1.04 | 0.200  ±0.030 | 0.0313  ±0.0047 | 0.158  ±0.024 | 6.50  ±0.98 | 5.21  ±0.78 | 274  ±41 | 6.96  ±1.04 | 9.75  ±1.46 |  |
| S6H100 | 5.37  ±0.54 | 2.01  ±0.20 | 3.81  ±0.57 | 11.9  ±1.8 | 0.182  ±0.027 | 0.0292  ±0.0044 | 0.165  ±0.025 | 11.0  ±1.7 | 5.11  ±0.77 | 258  ±39 | 6.60  ±0.99 | 7.47  ±1.12 |  |
| S24H40 | 7.38  ±0.74 | 2.32  ±0.23 | 1.99  ±0.30 | 4.14  ±0.62 | 0.164  ±0.025 | 0.0348  ±0.0052 | 0.160  ±0.024 | 3.30  ±0.50 | 5.68  ±0.85 | 320  ±48 | 8.80  ±1.32 | 12.3  ±1.8 |  |
| S24H60 | 6.63  ±0.66 | 2.34  ±0.23 | 2.57  ±0.39 | 7.43  ±1.11 | 0.221  ±0.033 | 0.0304  ±0.0046 | 0.155  ±0.023 | 6.40  ±0.96 | 4.69  ±0.70 | 276  ±41 | 6.58  ±0.99 | 9.90  ±1.49 |  |
| S24H100 | 5.17  ±0.52 | 1.96  ±0.20 | 3.80  ±0.57 | 12.3  ±1.8 | 0.192  ±0.029 | 0.0292  ±0.0044 | 0.168  ±0.025 | 11.4  ±1.7 | 6.61  ±0.99 | 257  ±39 | 6.80  ±1.02 | 8.00  ±1.20 |  |

**Table S3.** *Analysis of selected physicochemical properties of fertilizers*

| Materials | Properties | | | |
| --- | --- | --- | --- | --- |
|  | **Viscosity** | **Dry mass** | **Density** | **pH** |
|  | *mPa⋅s* | *%* | *kg/m^3^* |  |
| LFM | 39.7 | 21.6 | 1.14 | 4.3 |
| LF | 61.8 | 21.3 | 1.12 | 4.6 |
